# Protein-protein interaction-based high throughput screening for adenylyl cyclase 1 inhibitors: Design, implementation, and discovery of a novel chemotype

**DOI:** 10.3389/fphar.2022.977742

**Published:** 2022-09-06

**Authors:** Tiffany S. Dwyer, Joseph B. O’Brien, Christopher P. Ptak, Justin E. LaVigne, Daniel P. Flaherty, Val J. Watts, David L. Roman

**Affiliations:** ^1^ Department of Medicinal Chemistry and Molecular Pharmacology, College of Pharmacy, Purdue University, West Lafayette, IN, United States; ^2^ Department of Pharmaceutical Sciences & Experimental Therapeutics, The University of Iowa College of Pharmacy, Iowa City, IA, United States; ^3^ Nuclear Magnetic Resonance Facility, The University of Iowa Carver College of Medicine, Iowa City, IA, United States; ^4^ Iowa Neuroscience Institute, Iowa City, IA, United States; ^5^ Purdue Institute for Drug Discovery, West Lafayette, IN, United States; ^6^ Purdue Institute for Integrative Neuroscience, West Lafayette, IN, United States; ^7^ Holden Comprehensive Cancer Center, The University of Iowa Hospitals & Clinics, Iowa City, IA, United States

**Keywords:** inflammatory pain, adenylyl cyclase, cAMP signaling, high throughput screen (HTS), Ca2+, calmodulin (CAM), drug discovery

## Abstract

Genetic and preclinical studies have implicated adenylyl cyclase 1 (AC1) as a potential target for the treatment of chronic inflammatory pain. AC1 activity is increased following inflammatory pain stimuli and AC1 knockout mice show a marked reduction in responses to inflammatory pain. Previous drug discovery efforts have centered around the inhibition of AC1 activity in cell-based assays. In the present study, we used an *in vitro* approach focused on inhibition of the protein-protein interaction (PPI) between Ca^2+^/calmodulin (CaM) and AC1, an interaction that is required for activation of AC1. We developed a novel fluorescence polarization (FP) assay focused on the PPI between an AC1 peptide and CaM and used this assay to screen over 23,000 compounds for inhibitors of the AC1-CaM PPI. Next, we used a cellular NanoBiT assay to validate 21 FP hits for inhibition of the AC1-CaM PPI in a cellular context with full-length proteins. Based on efficacy, potency, and selectivity for AC1, hits **12**, **13**, **15**, **18**, **20**, and **21** were prioritized. We then tested these compounds for inhibition of AC1 activity in cyclic AMP (cAMP) accumulation assays, using HEK293 cells stably expressing AC1. Hit **15** contained a dithiophene scaffold and was of particular interest because it shared structural similarities with our recently reported benzamide series of AC1 inhibitors. We next tested a small set of 13 compounds containing the dithiophene scaffold for structure-activity relationship studies. Although many compounds were non-selective, we observed trends for tuning AC1/AC8 selectivity based on heterocycle type and substituents. Having an ethyl on the central thiophene caused the scaffold to be more selective for AC8. Cyclization of the alkyl substituent fused to the thiophene significantly reduced activity and also shifted selectivity toward AC8. Notably, combining the fused cyclohexane-thiophene ring system with a morpholine heterocycle significantly increased potency at both AC1 and AC8. Through designing a novel FP screen and NanoBiT assay, and evaluating hits in cAMP accumulation assays, we have discovered a novel, potent, dithiophene scaffold for inhibition of the AC1- and AC8-CaM PPI. We also report the most potent fully efficacious inhibitor of AC8 activity known to-date.

## Introduction

Adenylyl Cyclase Type 1 (AC1) is a promising target for treating chronic inflammatory pain. The overlapping neuronal expression of membrane-bound AC proteins prioritizes obtaining selectivity when targeting a single isoform (Dessauer et al., 2017). Studies with adenylyl cyclase knockout (KO) mice further emphasize this point. In mice lacking AC1, behavioral responses to inflammatory stimuli were nearly abolished, and memory deficits were nearly absent. Conversely, AC1/AC8 double KO mice exhibited reduced behavioral responses to inflammatory stimuli as expected but also displayed significant memory deficits (Ferguson & Storm, 2004; Liauw et al., 2005; Chen et al., 2014). Moreover, chronic inflammatory pain stimuli increases AC1 activity in mice, while AC1 KO mice had significantly less chronic pain (Wei et al., 2002; Vadakkan et al., 2006; Corder et al., 2013). Studies have also reported that pharmacologic inhibition of AC1 also elicits a reduction in responses to inflammatory pain stimuli (Wang et al., 2011; Brust et al., 2017; Kaur et al., 2019; Scott et al., 2022). The combination of these findings support AC1 as a promising target for treating chronic inflammatory pain conditions; however, they also emphasize the importance of selectively targeting AC1 over other AC isoforms, most notably AC8, which is co-localized in the same neuronal tissues (Defer et al., 2000).

AC1 signaling centers around its ability to synthesize the second-messenger cyclic adenosine monophosphate (cAMP) from ATP, and this primary function of AC1 is regulated by several signaling proteins. Gα_i/o_ and Gβγ subunits inhibit the activity of AC1, whereas Gα_s_ and Ca^2+^/calmodulin (CaM) stimulate AC1 activity, with Ca^2+^/CaM exhibiting particularly robust stimulation (Sadana & Dessauer, 2009; Dessauer et al., 2017). Therefore, inhibition of the AC1/CaM protein-protein interaction (PPI) would, in theory, inhibit Ca^2+^/CaM-mediated activation of AC1, thus diminishing cAMP production mediated through AC1. AC8 is also stimulated by Ca^2+^/CaM, although the binding interactions between the Ca^2+^/CaM complex with AC8 differs from how it interacts with AC1. Specifically, Ca^2+^/CaM interacts with the C1b regulatory domain of AC1 to activate the enzyme, but interacts with both the N-terminal and AC8-C2b regulatory domains of AC8 for activation (Simpson et al., 2006; Willoughby & Cooper, 2007; Masada et al., 2009; Mou et al., 2009; Masada et al., 2012; Herbst et al., 2013). These differences in Ca^2+^/CaM binding interactions between the isoforms provides the basis for developing a novel biochemical fluorescence polarization assay that focuses on the CaM binding regions of AC1 and AC8. Additionally, the different interactions of AC1 and AC8 with CaM may provide a basis for selectively targeting inhibition of the AC1-CaM PPI in a more focused approach. The research described here provides evidence that this focused approach, which incorporates small AC peptides correlated to the respective CaM binding domains can identify compounds with novel chemotypes capable of selectively inhibiting the full-length AC/CaM PPIs. Here we report the screening design, implementation, and characterization of hit compounds from this high-throughput screen. In addition, we show the characterization of initial hits in terms of binding, via protein NMR, cell-based protein-protein interaction inhibition, and selectivity between adenylyl cyclase 1 and adenylyl cyclase 8. Furthermore, we present additional cell-based cAMP accumulation data for a novel dithiophene scaffold discovered through our screen as well as preliminary structure-activity relationship (SAR) studies. Collectively, this demonstrates the utility and robustness of these methods to identify novel inhibitor scaffolds that target the AC/CaM PPIs.

## Materials and methods

### DNA cloning

The human CaM (hCaM) bacterial expression vector was constructed as previously described (Hayes et al., 2018). Briefly, the protein-coding sequence of hCaM (residues 1–149, Addgene plasmid #47598) was cloned into pET-His6-GST-TEV-LIC (Addgene plasmid #29655) using ligation independent cloning. Then, this bacterial expression vector was used to generate hCaM with an N-terminal 6X-His-GST tag with a tobacco etch virus (TEV) protease recognition site between the GST and CaM regions.

The NanoBiT PPI TK/BiT MCS vectors were purchased from Promega (Madison, WI) for cell-based NanoBiT experiments. The hCaM residues 1–149, residues 1–1,248 of rat AC8 (rAC8), and the human AC1 (hAC1) residues 1–1,118 were cloned into the NanoBiT vectors per manufacturers’ protocols, using the restriction sites NheI/XhoI for hCaM and BglII/XhoI. All DNA sequences were confirmed with Sanger sequencing (Iowa Institute of Human Genetics, Iowa City, IA).

### Protein purification

hCaM was expressed and purified as described previously with minor modifications (Hayes et al., 2018). Briefly, hCaM was transformed into BL21-CodonPlus (DE3)-RIPL *E. Coli* and incubated at 37°C with shaking at 275–300 RPM until the OD_600_ reached 2.0. The bacterial culture was placed on ice with gentle mixing before protein production induction with isopropyl-β-D-1-thiogalactoside (RPMI) at a final concentration of 1.0 mM. Protein production continued for an additional 16 h at 18°C with shaking at 300 RPM. The bacterial culture was pelleted and resuspended in 50 ml of resuspension buffer (50 mM Tris pH 8.0, 150 mM NaCl, 10 mM imidazole, and a protease inhibitor cocktail consisting of leupeptin, E64, and phenylmethylsulphonyl fluoride) per 1 L of original bacterial culture. The resuspended bacterial cells were then subjected to a freeze-thaw cycle using LN_2_. The resuspended cells were thawed, supplemented with 1 mg/ml chicken egg lysozyme (Sigma-Aldrich, St. Louis, MO), and agitated on a tube rotator for 1 h before being subjected to two more freeze-thaw cycles with LN_2_. After cell lysis was complete, 100 μg DNase (Roche) was added to reduce viscosity and prepare the sample for centrifugation. The lysed cells were then subjected to ultracentrifugation at 100,000 x g to clear the lysate from the supernatant. The separated supernatant was purified as described previously with minor modifications (Hayes et al., 2018). Briefly, using an AKTA FPLC (GE Life Sciences, Chicago, IL) the lysate was loaded onto NIS6FF resin and purified using a gradient elution over 20 column volumes of resuspension buffer with 400 mM imidazole. Peak fractions were pooled, diluted in 50 mM Tris pH 7.5, 1 mM CaCl_2_, and loaded onto a glutathione sepharose 4FF column (GE Life Sciences, Chicago, IL). Purified GST-CaM was eluted using a gradient elution over 10 column volumes of 50 mM Tris pH 7.5, 1 mM CaCl_2,_ and 10 mM glutathione. Peak fractions were collected and analyzed with SDS-PAGE gel electrophoresis to assess their purity. Fractions greater than 90% pure were pooled and exhaustively dialyzed against 20 mM HEPES pH 7.4, 100 mM KCl, at 4°C to remove Ca^2+^, before samples were aliquoted, flash-frozen in LN_2_, and stored at −80°C. To purify tag-free CaM, we incubated GST-CaM with His-tagged TEV protease at a 20:1 M ratio (GST-CaM/TEV) while dialyzing the sample against 20 mM HEPES pH 7.4, 100 mM KCl, 1 mM CaCl_2_. Dialysis and TEV treatment proceeded overnight at 4°C. The TEV treated CaM sample was then loaded onto an IMAC to separate 6XHis-tagged GST and TEV from CaM. The resulting flow-through fractions were collected and analyzed with SDS-PAGE and Coomassie staining. Fractions determined to be 95% pure CaM were concentrated to >10 mg/ml using an Amicon centrifugal concentrator with a 3kD cutoff (Amicon, EMD Millipore, Billerica, MA). Samples of pure CaM were aliquoted, flash-frozen in LN_2_, and stored at -80°C. Production of ^15^N-labeled CaM for NMR experiments was accomplished as described above, with the only modification being that bacterial culturing was completed in M9 minimal media supplemented with 1 g of ^15^NH_4_Cl per L.

### Fluorescence polarization assays

Peptides for adenylyl cyclase proteins, AC1 and AC8, were generated based on the residues or domains known to interact with CaM (Vorherr et al., 1993; Levin & Reed, 1995; Masada et al., 2012). The peptide corresponding to residues 492–520 of human AC1 was obtained from Genemed Synthesis (San Antonio, Texas). Peptides corresponding to human AC8 residues 30–54 (N-Terminal) and 1,191–1,214 (C2b regulatory domain) were purchased from Genscript (Piscataway, NJ). Each peptide was designed to contain an additional N-terminal residue labeled with the fluorophore Cy5. This additional residue for the AC8 peptides was cysteine, while the AC1 peptide has an additional isoleucine. The FP experiments were completed in black 384-well microplates (Corning #3575), and fluorescence was monitored using a BioTek Synergy 2 (Winooski, VT) equipped with excitation 620/40 nm and emission 680/30 nm filters, and a 660 nm dichroic mirror with polarizers. The addition of individual components was done as follows. First, 20 μl of 3X test compound in FP assay buffer (20 mM HEPES pH 7.4, 100 mM KCl, and 50 μM CaCl_2_), 3X calmidazolium (CDZ) in FP assay buffer, or 3X DMSO in FP assay buffer was added to their respective wells. Next, 20 μl of 3X GST-CaM in FP assay buffer was added to all wells, and the plate was incubated at room temperature for 30 min. Lastly, 20 μl of 3X AC peptide in FP assay buffer was added to all wells, and the plate was incubated in the dark at room temperature for either the amount of time indicated for the assay optimization experiments or 2 h for the high-throughput screening experiments. Fluorescence was then measured, and the polarization output was calculated using the measured intensities of emitted light parallel and perpendicular to the excitation light, shown in Equation 1 below. The final concentrations for all Cy5-labeled AC peptides (AC1 C1b, AC8 Nt, AC8 C2b) were 100 nM, the final concentration of CDZ was 100 μM, and the final concentration of DMSO was 0.15–1%, depending on the compound library that was being tested. Data represents a mean of three independent experiments (N = 3) ± SEM for potency and selectivity profiling for AC-CaM FP. Data were analyzed using GraphPad Prism 8 and TIBCO Spotfire.

$$Polarization=\frac{Intensity_{parallel}-Intensity_{perpendicular}}{Intensity_{parallel}+Intensity_{perpendicular}}$$ (1)

### FDA-approved library FP screen

The FDA-approved library (Selleck Chemical, Houston, TX) was screened at a final concentration of 14.5 μM (0.72% DMSO). On the day of the assay, a “middle” plate (ThermoFisher #264574) was created to simplify the addition of 20 μl of 3X compound to the final assay plate. First, using a Hamilton MircroLab Star liquid-handling robot, 2 μl of 2 mM compound from the FDA stock plate was added to the middle plate containing 90 μl of FP assay buffer. Next, 20 μl of 3X compound from the middle plate was added to the assay plate (Corning #3575). Controls were then added to empty wells (32 wells DMSO control, 32 wells CDZ positive control). Next, 20 μl of 3X GST-CaM (100 nM GST-CaM final for the screen with AC1 C1b peptide) was added to the entire plate, and the assay mixture was incubated for 30 min at room temperature. Finally, 20 μl of 3X AC1 C1b Cy5 labeled peptide (100 nM final AC1 C1b) was added to the entire plate, and the assay mixture was incubated at room temperature in the dark for 2 h before polarization was measured as described above.

### Microsource spectrum collection FP screen

The Microsource spectrum collection (MMSP) (Microsource Discovery Systems, Gaylordsville, CT) was screened as outlined above for the FDA-approved library with some minor differences. First, the stock concentration of compounds was 10 mM. Therefore, the middle plate contains 1 μl of MMSP compounds with 90 μl of FP assay buffer (concentration of the compound in the middle plate: 109.8 μM). To achieve the desired 3X concentration in the test plate, 12 μl of FP assay buffer was added to the test plate before adding 8 μl of the compound from the middle plate (3X compound in test plate: 43.9 μM). After compounds were added to the test plates addition of controls, GST-CaM, and Cy5-labeled AC1 C1b peptide were completed as described for the FDA-approved library. The test plate’s final concentration of MMSP compounds was 14.64 μM (0.147% DMSO).

### ChemBridge DIVERSet library FP screen

The screening protocol for the ChemBridge DIVERSet library (San Diego, CA) of compounds as described for the FDA-approved library with the same minor differences as the MMSP library described above. The test plate’s final concentration of ChemBridge DIVERSet compounds was 14.64 μM (0.147% DMSO).

### ChemDiv CNS targeted library FP screen

For the ChemDiv CNS targeted library (ChemDiv, San Diego, CA) of compounds, the protocol is the same as that outlined for the FDA-approved library, with minor differences as the MMSP and ChemBridge DIVERSet libraries. The test plate’s final ChemDiv CNS targeted compounds’ concentration was 14.64 μM (0.147% DMSO).

### AC-CaM NanoBiT assays

The AC8/CaM NanoBiT assays were performed mainly as previously described (Hayes et al., 2018). The AC1/CaM NanoBiT assays were performed similarly to the AC8/CaM NanoBiT assays with some differences. Briefly, HEK293T cells were cultured in Gibco DMEM supplemented with 10% FBS (Gibco) and 1% Penicillin/Streptomycin at 37°C and 5% CO_2_. Cells were plated at 25,000 cells/well in 96-well half area plates (Corning #3885) pretreated with poly-d-lysine. Cells were cultured for 16 h before transfection with Lipofectamine 3,000 (ThermoFisher Scientific, Waltham, MA) and the designated AC-CaM NanoBiT DNA constructs described above. Transfections were performed according to the manufacturer’s protocol, and transfected cells were incubated for 40–44 h before compound treatment.

On the day of the assay, the culture medium was removed and replaced with 50 μl of 1X Compound, BAPTA-AM (50 μM final) (Sigma-Aldrich), or vehicle in HBSS (ThermoFisher Scientific, Waltham MA) supplemented with 20 mM HEPES pH 7.4. The assay plate was then incubated at 37°C for 30 min before 12.5 μl of 5X NanoGlo Live-cell substrate (Promega, Madison, WI), prepared according to the manufacturer’s protocol, was added to each well. Next, the plate was read on a BioTek Synergy 2 plate reader at 37°C in luminescence mode for 30 min to establish a baseline for the protein-protein interaction. Next, 12.5 μl of 6X Thapsigargin (ThermoFisher Scientific, Waltham, MA) was added to each well, and luminescence was read for an additional 60 min. The area under the curve (AUC) analysis quantified the AC-CaM association for vehicle, BAPTA-AM, and compound-treated cells. Using the AUC for compound-treated cells, the data were normalized to vehicle with 1 μM thapsigargin added (100%) and 50 μM BAPTA-AM (0%). Data were analyzed using GraphPad Prism 8, with each data set representing a mean of three independent experiments (N = 3) ± SEM.

### CellTiter-glo 2.0 cell viability assay

Cell viability measurements were performed using a BioTek Synergy 2 plate reader luminescence mode. HEK293T cells were cultured in Gibco DMEM supplemented with 10% FBS (Gibco) and 1% Penicillin/Streptomycin at 37°C and 5% CO_2_. Cells were plated at 25,000 cells/well in 96-well half area plates (Corning #3885) coated with poly-d-lysine and allowed to incubate for 40–44 h. On the day of the assay, culture media was aspirated and replaced with 25 μl of 2X compound in HBSS supplemented with 20 mM HEPES pH 7.4 or 2X vehicle (HBSS supplement with 20 mM HEPES pH 7.4). After adding the compound or vehicle, the plate was incubated at 37°C for 30 min. Next, 25 μl of 2X CellTiter-Glo 2.0 reagent (Promega, Madison, WI) was added, and the plate was mixed on an orbital shaker for 30 min to induce cell lysis and equilibrate the plate to room temperature. Luminescence was then measured for 30 min to establish a stable signal. The final three luminescence values from the 30-min read were averaged and used to assess cell viability. Cell viability for tested compounds was normalized relative to vehicle (100%) and wells containing buffer with no cells (0%). Data represent the mean of three independent experiments (N = 3) ± SEM.

### cAMP accumulation assay

Cryopreserved HEK293 cells with AC3 and AC6 knocked out, and stably expressing AC1, AC2, AC5, or AC8 (AC1-HEK Δ3/6 KO, AC2-HEK Δ3/6 KO, AC5-HEK Δ3/6 KO, or AC8-HEK Δ3/6 KO cells, respectively; Soto-Velasquez et al., 2018) were plated at 5,000 cells/well. Cells were then incubated for 1 h at 37°C and 5% CO_2_. Cells were then treated with varying concentrations of inhibitors for 30 min, followed by treatment with calcium ionophore A23187 (3 μM) in 500 μM 3-isobutyl-1-methylxanthine (IBMX), a phosphodiesterase inhibitor, for 1 h at RT (Sigma Aldrich, St. Louis, MO). Finally, Cisbio HTRF detection reagents were added and incubated for 1–24 h at RT. Plates were read according to manufacturer’s protocol on an HTRF-compatible plate reader at 620 and 665 nm cAMP concentrations were interpolated from a cAMP standard curve. Data represents the mean ± SEM of IC_50_ values or percent of maximum A23187 inhibition from the mean of three independent experiments (N = 3). For cytotoxicity assays, compounds were tested at 30 μM and this protocol was used with the exception of detection reagents; CellTiter-Glo 2.0 reagent was used according to manufacturer’s protocol and luminescence was read on the Synergy Neo2 plate reader. Data are normalized to cells treated with vehicle (100%) or 2% tween (0%) and are represented as the percent of vehicle, from the average of three independent experiments (N = 3).

### Nuclear magnetic resonance

All NMR samples contained 100 μM ^15^N-CaM in 20 mM HEPES pH 7.4, 100 mM KCl, 10 mM CaCl2, 10% D_2_O, and 5% DMSO. Initial spectra were collected for CaM alone before individual compounds were titrated to observe chemical shift perturbations. All spectra were acquired at 298 K using a 600 MHz Bruker Avance NEO NMR spectrometer with a triple resonance gradient cryoprobe. NMR spectra were processed using NMRPipe and analyzed using NMRFAM-SPARKY.

## Results

### Fluorescence polarization assays

**FIGURE 1:**
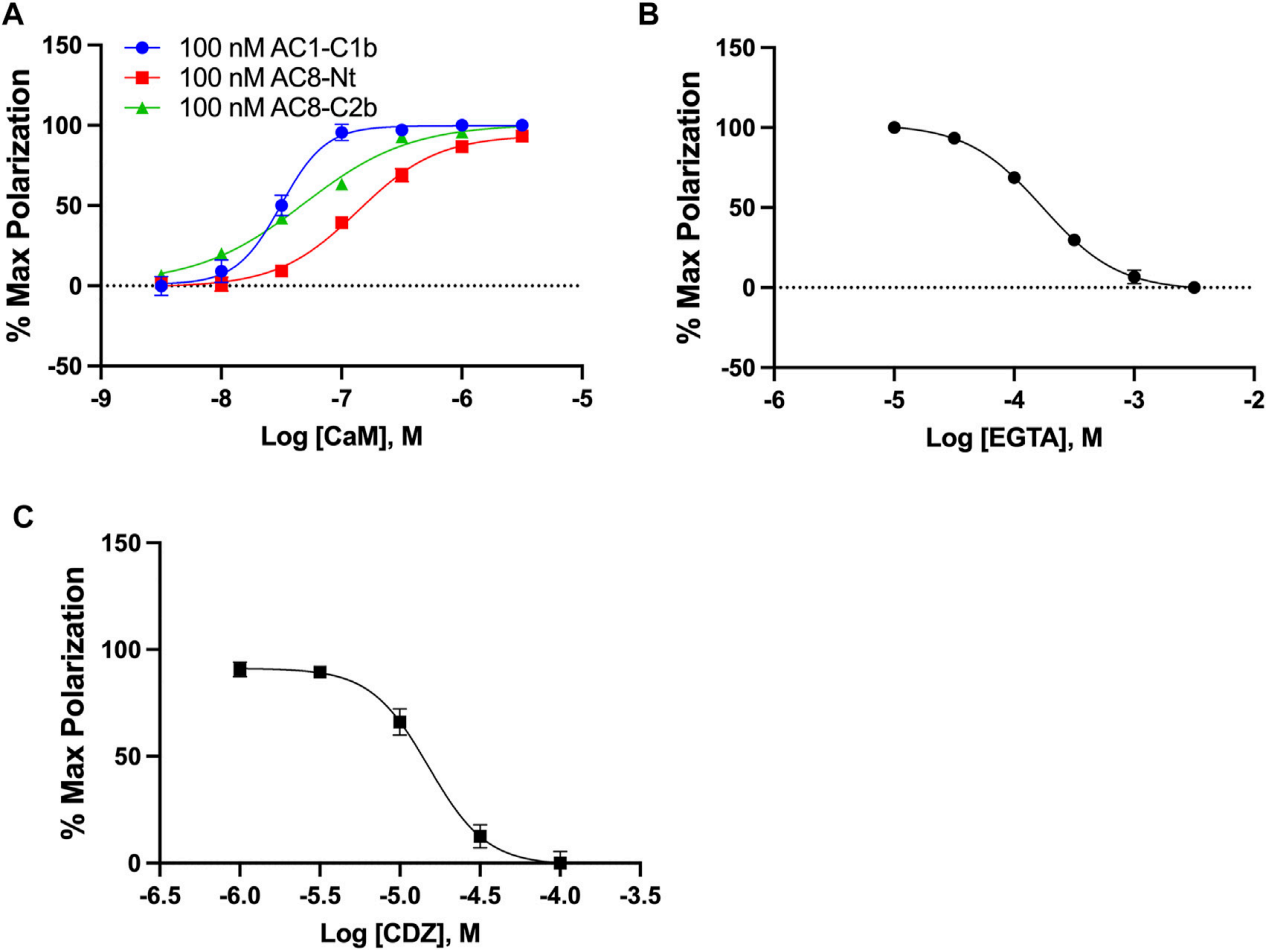
AC1/CaM FP Assay development. **(A)** 100 nM AC1-C1b (Blue), AC8-NT (Red), or AC8-C2b (Green) peptide with increasing concentrations of GST-CaM. Saturation is reached at ∼100 nM GST-CaM. **(B)** 100 nM AC1 peptide and 100 nM GST-CaM with increasing concentrations of EGTA. **(C)** CRC for CDZ with 100 nM AC1-C1b and 100 nM GST-CaM, with 1% DMSO in the buffer. The AC1/CaM IC_50_ value for CDZ was ∼15 μM and the IC_90_ was ∼100 μM, which was used in the final assay. Calmidazolium CAS number: 57265–65–3. All data represents n = 3 experiments ±SEM.

Intending to identify small molecule inhibitors that selectively target the AC1/CaM PPI, we developed and optimized an FP assay for a high-throughput screening (HTS) campaign. For simplicity, peptides will be denoted AC1-C1b, AC8-Nt, and AC8-C2b, all containing an N-terminal Cy5 fluorophore label. The FP assays for AC8-Nt and AC8-C2b, established previously (Hayes et al., 2018), were used as counter screens for compounds identified in the HTS campaign and served as a guide for AC1/CaM assay development. The assay buffer used in all FP experiments consisted of 20 mM HEPES pH 7.4, 100 mM KCl and 50 μM CaCl_2_. At fixed concentrations of peptide and increasing concentrations of CaM, the dissociation constants (K_d_) for AC1-C1b, AC8-Nt, and AC8-C2b were quantified to be 31.5, 136.5, and 48.5 nM, respectively (Figure 1A). The nature of CaM as a calcium-binding protein offered an opportunity to probe the calcium sensitivity of the AC1-C1b/CaM FP assay. Investigating the AC1-C1b/CaM interaction with EGTA (Ca^2+^ chelator) showed the interaction to be Ca^2+^ dependent, which was expected (Figure 1B). Incorporating EGTA as a positive control worked well for the AC8/CaM FP assay but did not provide a sufficient signal window for the AC1/CaM FP assays. Calmidazolium (CDZ), a compound known to bind CaM and previously identified in an AC8/CaM inhibitor screen, served as the positive control with an IC_50_ of 15.1 μM (Figure 1C) (Gietzen et al., 1981; Ahlijanian & Cooper, 1987; Lubker & Seifert, 2015). The AC1-C1b/CaM assay stability was assessed in the presence of 0–5% DMSO, with measurements taken at 1, 2, 4, and 6 h (Supplementary Figure S1 and Table 1). Throughout these experiments, Z′ values were calculated using Equation 2 (Zhang et al., 1999) shown below to assess the assay for rigor and reproducibility in an HTS campaign (Table 2). With a Z′ value cutoff of >0.65, the AC1-C1b/CaM FP assay tolerated up to 2.5% DMSO, and the signal was stable after 4 h of incubation at RT. The final assay conditions for the AC1-C1b/CaM are described in Table 1, with σ_p,n_ representing the standard deviation (SD) of positive and negative control, and μ_p,n_ representing the mean of the positive and negative control.

$$Z^{\prime }=1-\frac{\left(3\sigma_{p}+3\sigma_{n}\right)}{\left|\mu_{p}-\mu_{n}\right|}$$ (2)

**TABLE 1:** AC1/CaM FP assay conditions for the pilot screen. The table indicates the concentrations for all AC1/CaM FP assay components. The order of addition for the AC1/CaM FP assay was vehicle, compound, or the positive control (CDZ) followed by GST-CaM and AC1-C1b after a 30-min incubation at RT.

Final volume	GST-CaM	AC1-C1b	Vehicle	Positive control	% DMSO	Time
60 μl	100 nM (20 μl)	100 nM (20 μl)	FP buffer (20 μl)	100 μM CDZ (20 μl)<	1–2.5	2

**TABLE 2:** AC1/CaM FP pilot screen summary. The AC1/Cam FP pilot screen included four chemical libraries. The Z’ values are the ranges obtained for the designated libraries. A total of 54 validated hits were obtained from the pilot screen and through duplicate hits, unfavorable structures or potency/inhibition profiles a total of 25 compounds were advanced for concentration response curve assessment in the AC1/CaM and AC8/CaM FP assays.

Library	Compounds screened	Z′	Validated hits	Advanced to CRC
FDA-Approved	1,018	0.70–0.77	7	7
MMSP	2,320	0.69–0.77	13	7
ChemBridge DIVERSet	10,240	0.68–0.77	2	2
ChemDiv	7,680	0.69–0.76	32	9
**Total**	**21,258**	**∼0.69–0.77**	**54**	**25**

### AC1/CaM FP high throughput screen

A high-throughput screen of 21,258 compounds was completed using the AC1/CaM FP assay described above. Hit compounds identified in the initial HTS were verified by retesting with the primary AC1/CaM FP assay before advancing hit compounds to concentration-response curve (CRC) assessment in the AC1/CaM and AC8/CaM FP assays. Compounds were assessed based on their inhibition constants (K_i_), calculated using the Cheng-Prusoff equation (Equation 3) shown below (Cheng & Prusoff, 1973), and selectivity ratios for AC1 over the AC8 peptide interactions with CaM in the FP assays. In the equation below, K_i_ = The inhibitor constant, IC_50_ = observed IC_50_ in AC/CaM FP assay, K_d_ = AC/CaM dissociation constant, AC = Concentration of AC peptide.

$$Cheng-PrusoffEquation\;K_{i}=\frac{IC_{50}}{1+\frac{\mathrm{\lfloor A}\mathrm{C\rfloor}\;}{K_{d}}}$$ (3)

### FDA-approved library FP screen: AC1/CaM

**FIGURE 2:**
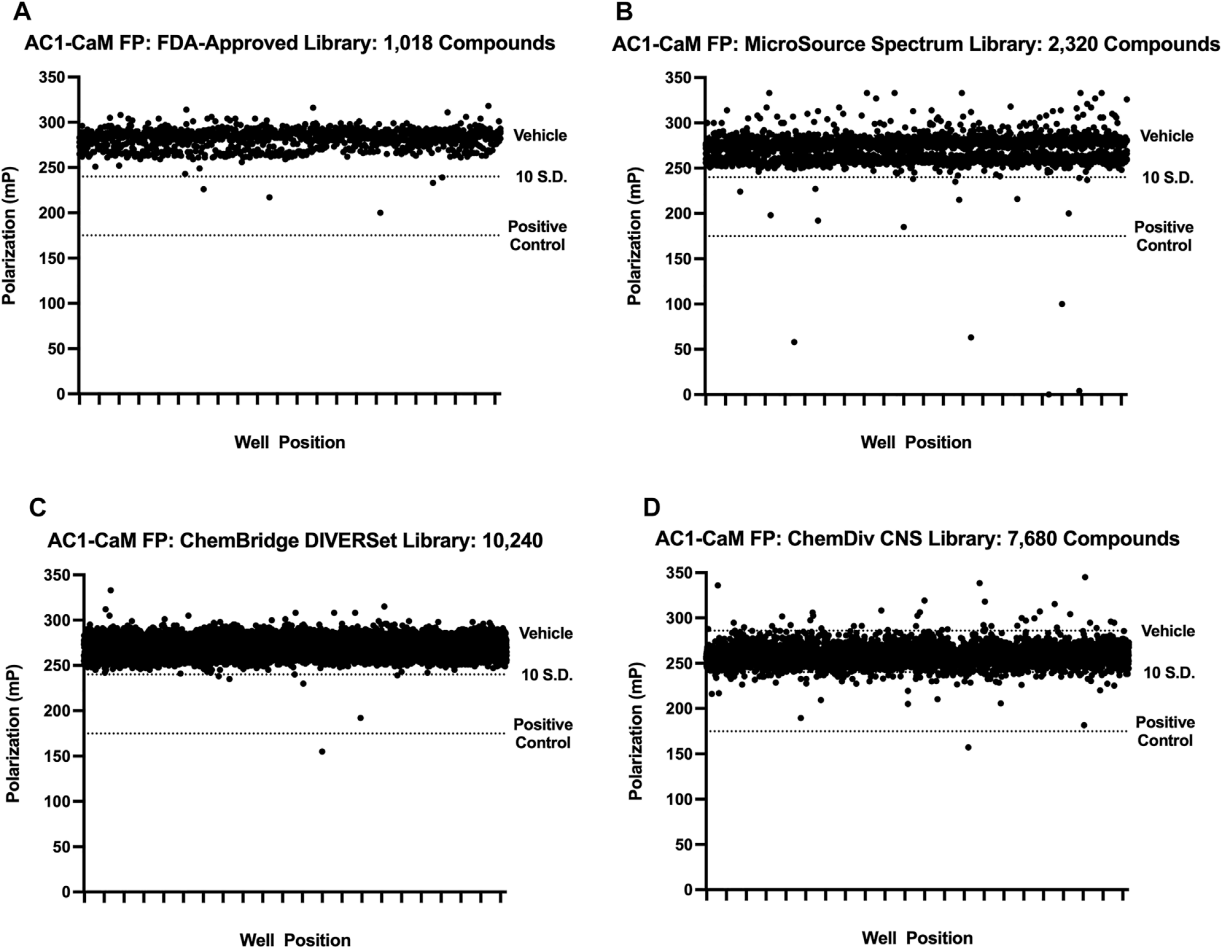
Pilot screen individual library results. All pilot screen assays used concentrations of AC1 and CaM shown in Table 1. The dotted line representing the vehicle is beneath the cluster of compounds screened (black circles). 100 μM CDZ is shown as the dotted line labeled as positive control. Polarization values correspond to well positions for plates within the library tested. **(A)** FDA-approved library, **(B)** MicroSource Spectrum (MMSP) library, **(C)** ChemBridge DIVERSet library, **(D)** ChemDiv CNS library.

First, a pilot screen was performed on 1,018 compounds from the FDA-approved library (Selleck Chemical, Houston, TX) using the AC1/CaM FP assay. Compounds were screened individually at a single concentration of 14.5 μM. The results of screening the FDA-approved library are shown in Figure 2A and summarized in Table 2. The calculated Z’ remained above the lower threshold for an acceptable HTS screen (>0.5) (Zhang et al., 1999), with Z′ ranging from 0.70–0.77. Hit criteria was established at compounds that exhibited a signal 5 SD below the mean value of the vehicle control (1% DMSO). This cutoff corresponded to ∼30% inhibition relative to 100 μM CDZ (100% inhibition) positive control. Finally, reproducibility experiments were conducted to assess assay variability over multiple screening days. The results of the variability testing showed that the compounds identified as hits on 1 day were identified again on the next day of screening (Supplementary Figure S2). Seven hits were validated at the screening concentration (14.5 μM), tested in triplicate. The hit rate for molecules that met the hit criteria and were validated as hits for the FDA-approved compounds was 0.7%.

### Microsource spectrum collection FP screen: AC1/CaM

We screened 2,320 compounds from the Microsource Spectrum Collection (MMSP, Gaylordsville, CT) at a single concentration of 14.6 μM. Hits were identified using a 5 SD. below the mean of the vehicle control (1% DMSO) cutoff and validated with a single concentration in the AC1/CaM FP assay as outlined for the FDA-Approved library. The results of screening the MMSP library are shown in Figure 2B and summarized in Table 2. The Z′ ranged from 0.69–0.77 for the MMSP library screen, and 13 compounds were validated as hits, yielding a hit rate of 0.56%. However, due to library duplication, three of the validated hits from the FDA-Approved library were re-identified as hits in the MMSP library (Mitoxantrone HCl, Alexidine, and Thonzonium). Interestingly, this served as a validation of these compounds as hits and as additional evidence for the reliability of this HTS assay. In addition, three of our initial hit compounds contained structures that were deemed unfavorable for optimization or further characterization (Chicago Sky blue, Protoporphyrin IX, and Chlorophyllide Cu complex) and were therefore excluded from further testing.

### ChemBridge DIVERSet library FP screen: AC1/CaM

The ChemBridge DIVERSet Library (ChemBridge Corp, San Diego, CA) is comprised of a compound collection that has been stringently filtered *in silico* to retain only compounds with desirable drug-like properties (MW ≤ 500, cLogP ≤5, H-Bond acceptors ≤10, H-Bond donors ≤5). A total of 10,240 compounds were screened and hits identified using the same 5 SD. Hits were validated with a single concentration in the AC1/CaM FP assay as outlined for the FDA-Approved library. The results of screening the ChemBridge DIVERSet library can be seen in Figure 2C, and the summary of the results is shown in Table 2. The Z′ for the ChemBridge DIVERSet library screen ranged from 0.68–0.77. Two compounds were validated as hits, yielding a hit rate of 0.02%.

### ChemDiv CNS targeted library FP screen: AC1/CaM

The ChemDiv CNS targeted library (CDI, ChemDiv, San Diego, CA) is comprised of compounds selected for CNS protein targets (98 protein sub-families/unit targets) based on literature searches (ranging from 2014-present) as well as novel X-ray and Cryo-EM structures deposited in the PDB. These searches are designed to identify chemical structures and substructures more likely to penetrate the blood-brain barrier due to their physicochemical properties and known activities. The criteria for evaluating the screening data produced with the CDI library were the same as for the previous libraries screened. 7,680 compounds from the CDI library were screened against the AC1/CaM FP assay. The results of screening the CDI library are shown in Figure 2D and the summary of the results is shown in Table 2. The Z′ ranged from 0.69–0.76, and hits were identified using the 5 SD cutoff and validated with a single concentration in the AC1/CaM FP assay as outlined for the FDA-Approved library. Screening the CDI library against the AC1/CaM FP assay yielded 32 validated hits, with a hit rate of 0.42%. To evaluate the potency of initial hits, concentration-response curves in the AC1/CaM FP assay were generated for the validated hits using the CDI library stocks. Of the 32 validated CDI hits, 9 compounds were advanced to the next phase of evaluation based on their magnitude of inhibition and K_i_ values for the AC1/CaM FP assay.

### Characterization of HTS hits: AC1/CaM and AC8/CaM FP

**FIGURE 3:**
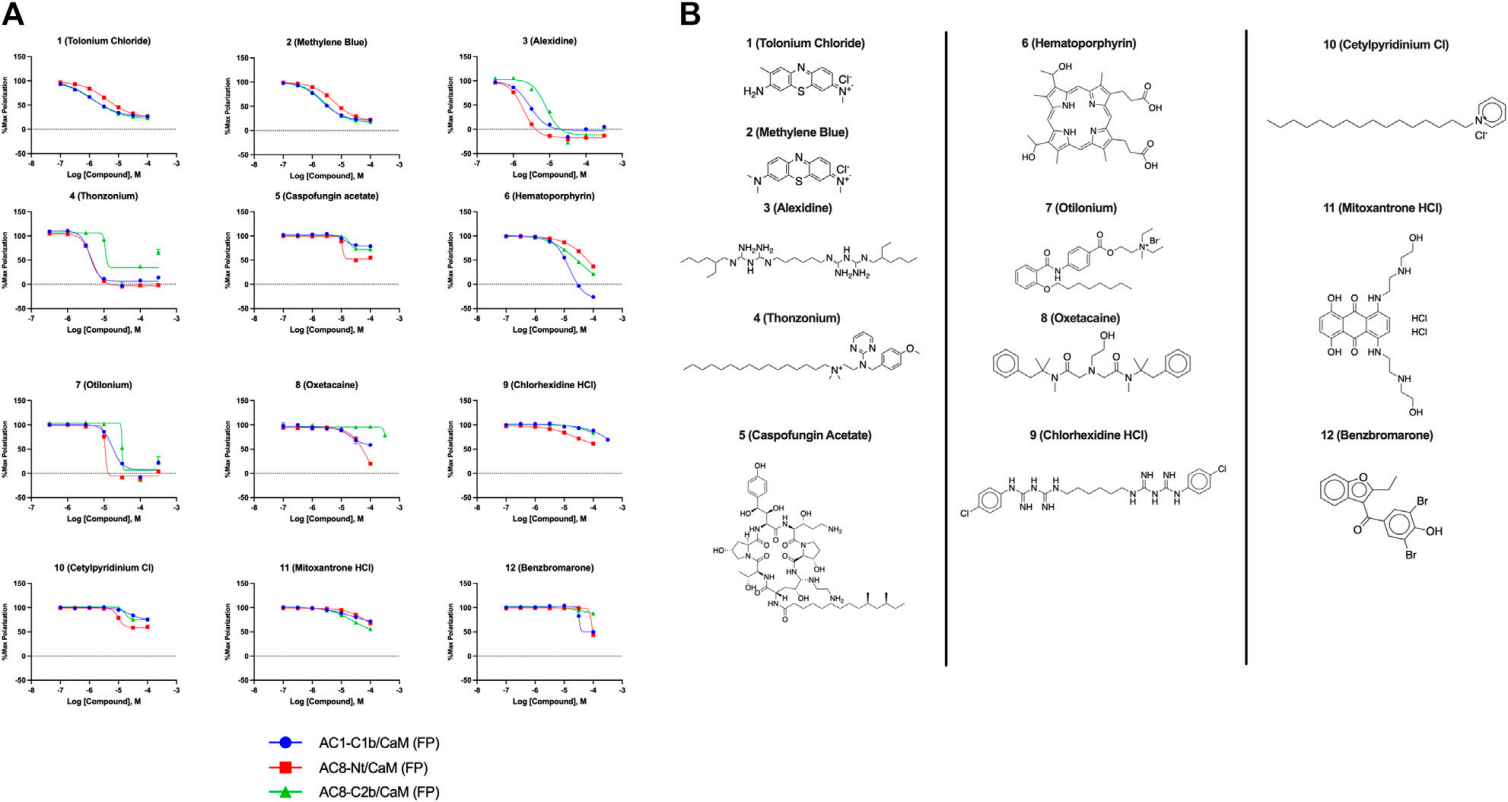
Concentration response curves **(A)** and structures **(B)** for FDA-approved and MMSP library hits. Each hit was used to generate CRCs in the AC1-C1b/CaM (blue circles), AC8-Nt/CaM (red squares), and AC8-C2b/CaM (green triangles) FP assays. **(B)** The structures for each hit are shown with the hit number and the given compound name. Data is normalized to vehicle (100% maximum polarization) and 100 μM CDZ (0% polarization). All data represents n = 3 experiments ±SE.M.

**FIGURE 4:**
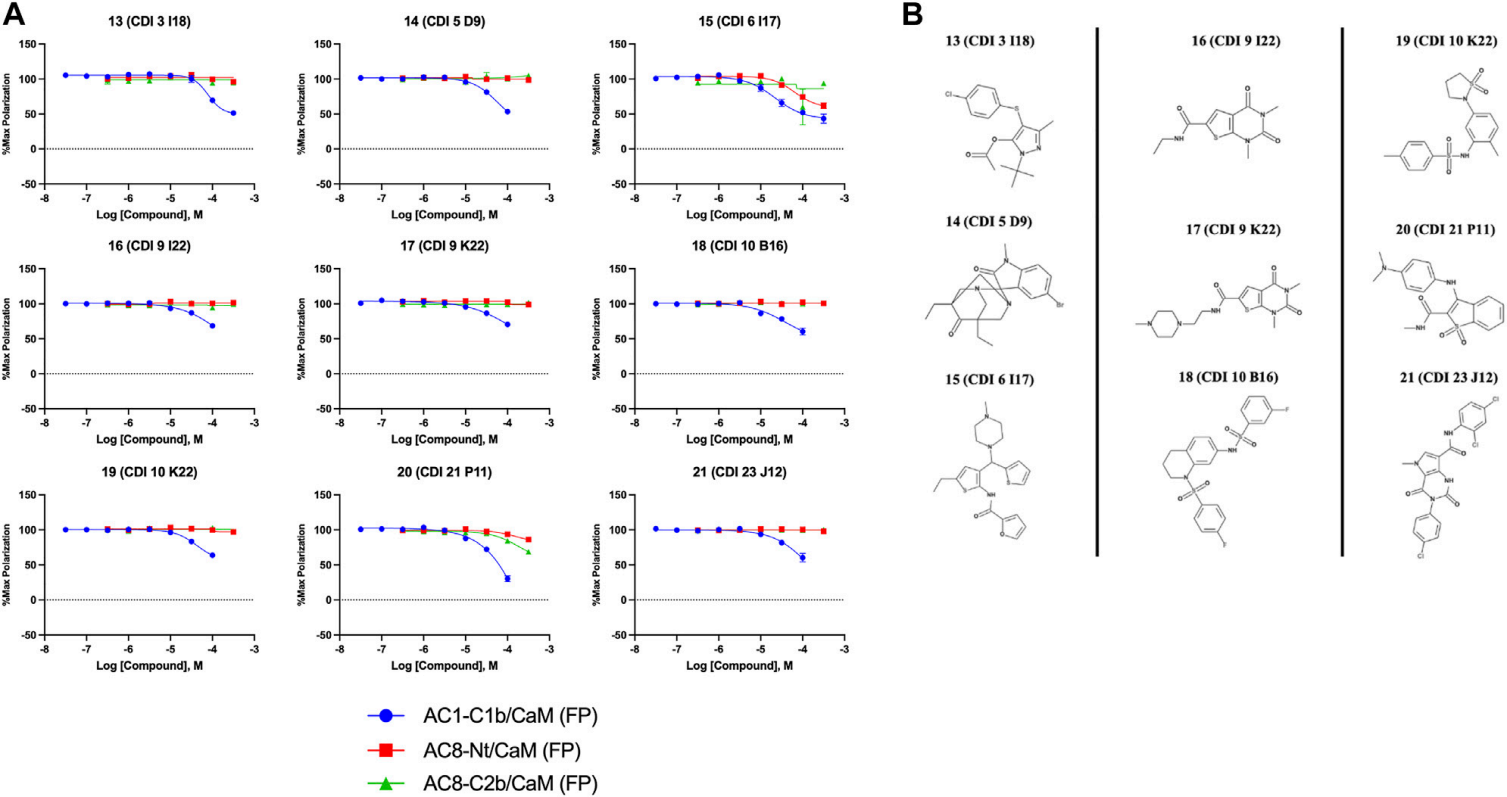
Concentration response curves **(A)** and structures **(B)** for the ChemDiv library hits. Each hit was used to generate CRCs in the AC1-C1b/CaM (blue circles), AC8-Nt/CaM (red squares), and AC8-C2b/CaM (green triangles) FP assays. **(B)** The structures for each hit are shown with the hit number and the given compound name. Data is normalized to vehicle (100% Maximum polarization) and 100 μM CDZ (0% polarization). All data represents n = 3 experiments ±SEM.

In total, 21,258 compounds from four compound libraries were screened in the AC1/CaM FP assay. From these screens, 25 validated hits were repurchased as a powder for CRC analysis. CRCs were generated for hits in the AC1/CaM primary assay and AC8/CaM counterscreen assays. Data were normalized to the vehicle (1% DMSO; 0% inhibition) and the 100 μM CDZ positive control (100% inhibition). However, as CDZ is not a complete inhibitor of the AC-CaM interaction, it did not abolish the FP signal altogether. As a result, several hit compounds inhibited the FP signal greater than 100%, using CDZ inhibition as a comparator. The CRCs and structures of the hit compounds for the FDA-approved, MMSP, and ChemBridge libraries are shown in Figure 3, and the CDI library hits are shown in Figure 4. The calculated AC1/CaM and AC8/CaM K_i_ values for all hit compounds are summarized in Table 3.

**TABLE 3:** AC1/CaM pilot screen CRC analysis. IC_50_ and % inhibition relative to 100 μM CDZ (100% inhibition) obtained for each compound in the AC1/CaM FP assay. The Ki values obtained with experimentally determined K_D_ values for each AC/CaM FP assay and the IC_50_ values obtained for each compound in the designated AC/CaM FP assay. The final two columns show the K_i_ ratios obtained for either AC8-Nt/CaM or AC8-C2b/CaM over AC1/CaM. Values with K_i_ ratios greater than 1 were more potent in the AC1/CaM FP assay over the designated AC8/CaM FP assay. All data represents n = 3 experiments, ± CI for AC1/CaM IC_50_ values.

#	Compound name	Structure	Compound library (Plate, well position)	AC1-CaM FP		Ki (μM)			Ki ratio (AC8 NT/AC1)	Ki ratio (AC8-C2b/AC1)
				IC_50_, μM (95% CI)	% inhib	AC1 C1b	AC8 NT	AC8 C2b		
1	Tolonium Chloride	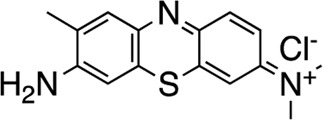	MMSP (Plate 5, N13)	1.24 (0.8–1.65)	75	0.29	2.3	0.44	7.9	1.5
2	Methylene Blue	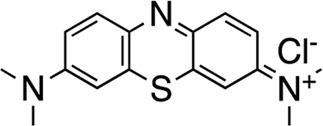	MMSP (Plate 3, D10)	2.18 (2.1–2.3)	80	0.51	3.2	0.82	6.3	1.6
3	Alexidine	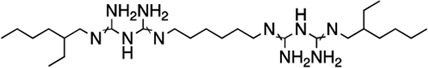	FDA-Approved (Plate 3, L11)	2.8 (2.4–3.3)	103	0.67	1.1	2.6	1.6	3.9
4	Thonzonium	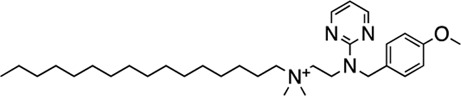	FDA-Approved (Plate 4, E17)	4.14 (1.3–4.6)	94	1.0	2.7	3.6	2.7	3.7
5	Caspofungin Acetate	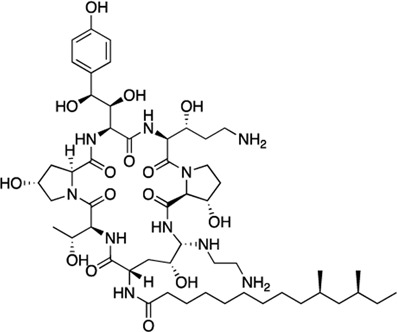	FDA-Approved (Plate 2, N18)	12.7 (10–15.7)	20	3.0	6.3	6.2	2.1	2.1
6	Hematoporphyrin	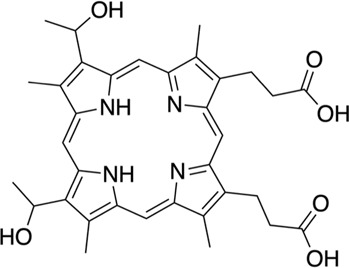	MMSP (Plate 6, O6)	14.7 (13.9–15.5)	131	3.5	61.7	10.1	17.8	2.9
7	Otilonium	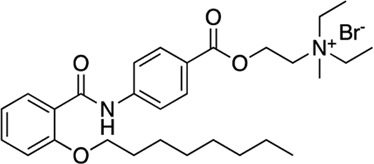	FDA-Approved (Plate 2, L16	17.1 (14.5–20)	92	4.1	6.3	10.3	1.6	2.5
8	Oxetacaine	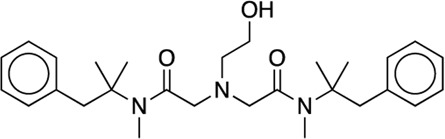	N/A	20 (11.5–32)	38	4.7	38	33.5	8.0	7.1
9	Chlorhexidine HCl	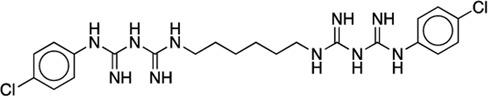	MMSP (Plate 1, P16)	22 (8.6–32)	15	5.2	13.8	20.8	2.7	4.0
10	Cetylpyridinium Cl	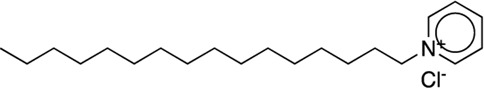	FDA-Approved (Plate 3, E18)	23.4 (16.1–31)	25	5.6	5.7	5.5	1.0	1.0
11	Mitoxantrone HCl	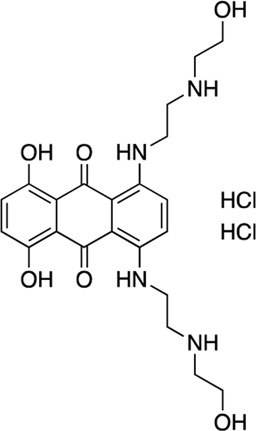		27.5 (17–42)	40	6.5	41.1	8.2	6.3	1.3
12	Benzbromarone	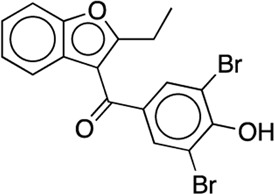		33 (17–54)	47	7.8	52.1	23.8	6.7	3.1
13	CDI 3 I18	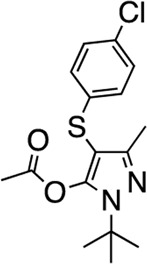		73.5 (Wide)	34	17.4	n/i	n/i	AC1 Selective	AC1 Selective
14	CDI 5 D9	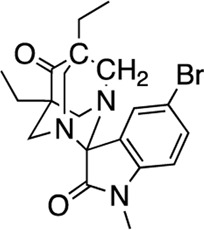		58.2 (34.3–80)	69	13.8	n/i	n/i	AC1 Selective	AC1 Selective
15	CDI 6 I17	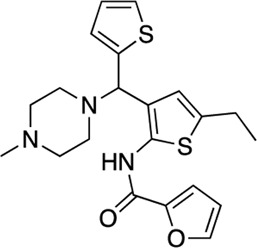		22.1 (14–43.4)	58	5.2	n/i	n/i	AC1 Selective	AC1 Selective
16	CDI 9 I22	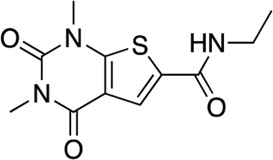		24.2 (8.5–48)	25	5.7	n/i	n/i	AC1 Selective	AC1 Selective
17	CDI 9 K22	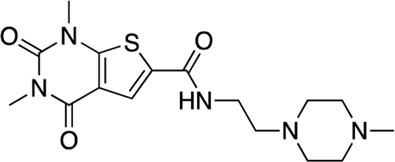		75.4 (19–132)	53	17.8	n/i	n/i	AC1 Selective	AC1 Selective
18	CDI 10 B16	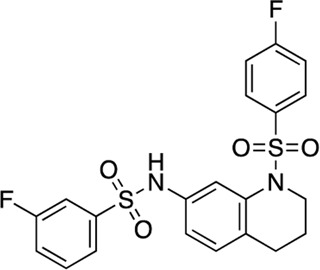		38.4 (15.1–52)	54	9.1	n/i	n/i	AC1 Selective	AC1 Selective
19	CDI 10 K22	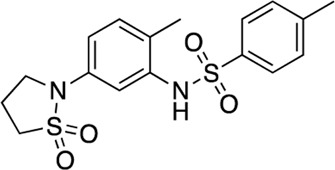		43.4 (27–59)	46	10.3	n/i	n/i	AC1 Selective	AC1 Selective
20	CDI 21 P11	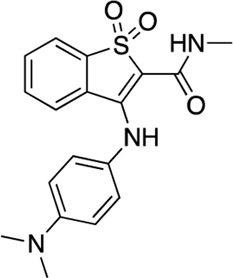		43 (Wide)	70	10.2	5.9	4.5	0.58	0.44
21	CDI 23 J12	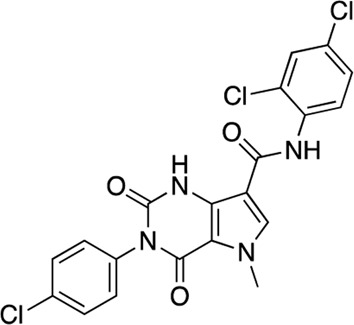		9.9 (5.8–13.2)	59	13.1	n/i	n/i	AC1 Selective	AC1 Selective

The most potent compound identified in the AC1/CaM FP screen was **1** (tolonium chloride) from the MMSP library, with a K_i_ of 0.29 μM for AC1-C1b/CaM (IC_50_ = 1.24 μM), 2.3 μM for AC8-Nt/CaM, and 0.44 μM for AC8-C2b/CaM. The second most potent hit, **2**, also from the MMSP library, has an almost identical structure to **1**, differing by the addition of a methyl group on the tricyclic scaffold. The K_i_ values for **2,** 0.51 μM for AC1-C1b/CaM (IC_50_ = 2.18 μM), 3.2 μM for AC8-Nt/CaM, and 0.82 μM for AC8-C2b/CaM, are nearly identical to those obtained for **1**. Further, **1** and **2** achieved 75–80% inhibition relative to the positive control (100 μM CDZ) in the AC1 and AC8/CaM FP assays at the highest concentration tested (100 μM). These two compounds did not exhibit a significant selectivity for the AC1/CaM interaction versus either AC8/CaM interaction, evidenced by similar K_i_ values. These compounds share a phenothiazine scaffold present in the known CaM binder, trifluoperazine (TFP, Stelazine™) and will be discussed below (Ahlijanian & Cooper, 1987; Hayes et al., 2018). It should also be noted that compound **2** (methylene blue) is a known nonspecific PPI inhibitor, although it was identified as a hit against AC1 (Chuang et al., 2022).

The next most potent hits were **3** and **4,** with K_i_ values of 0.67 and 1.0 μM for AC1-C1b/CaM (IC_50_ = 2.8 and 4.14 μM), respectively. Hits **3**, **4,** and **7** (K_i_ for AC1/CaM FP = 4.1 μM) were the duplicate hits identified in the FDA-approved and MMSP library screens. These three hits have been previously identified as AC8/CaM inhibitors (Hayes et al., 2018). Hits **3**, **4**, and **7** inhibited nearly 100% of the AC1/CaM FP signal; however, they exhibited only modest selectivity for AC1 over the AC8 constructs. The hits **5**, **6**, and **8–11** had K_i_ values ranging from 3.0–7.8 μM in the AC1/CaM FP assay. Hit **5** inhibited the AC1/CaM interaction with a K_i_ value of 3.0 μM in the FP assay and exhibited slight selectivity (2-fold) for AC1 over AC8 in the FP assays. Hit **6** inhibited the AC1/CaM FP assay to 131% relative to the positive control due to CDZ’s incomplete inhibition of the FP signal. Only hits **6** and **3** outperformed the positive control in the AC1/CaM FP assay. Hits **6** and **8** were the only non-CDI library compounds tested that preferentially inhibited AC1/CaM over AC8/CaM in the FP assays (Table 3; Figure 3). Hit **9** is structurally similar to **3** as both contain biguanidine scaffolds; however, **9** was a poor inhibitor of the AC1/CaM interaction in the FP assay, reaching only 15% inhibition, relative to the positive control, at the highest concentration tested.

The nine CDI CNS library hits **13**–**21** are from the original 32 confirmed hits from this library. After confirmation, we selected these nine hits for additional characterization based on the K_i_ and percent inhibition values gathered from CRCs in the AC1/CaM FP assay. In the FP assays, hits **13**–**19** and **21** exhibited selectivity for AC1/CaM over both AC8-Nt and AC8-C2b/CaM. The only exception was **20**, which was slightly more selective for AC8 over AC1 in the FP assays. The most potent CDI library hits in the AC1/CaM FP assay were **15** and **16,** with K_i_ values of 5.2 and 5.7 μM, respectively. However, **15** and **16** were less efficacious, achieving only 58 and 25% inhibition of the AC1/CaM PPI in the FP assay, less than several more potent hits from the FDA-Approved or MMSP libraries. From the CDI CNS library, the maximal inhibition in the AC1/CaM FP assay was ∼70% for **20** and **14**, with three compounds displaying less than 50% inhibition (**13**, **16**, and **19**). However, we placed an equal priority on potent compounds and those that exhibit selectivity for inhibition of AC1-CaM over AC8-CaM. We, therefore, moved forward with 21 (Table 3) of the original 25 hits identified in the pilot screens for assessment with full-length proteins in the NanoBiT assay.

### NanoBiT assay development: AC1/CaM

We used the NanoBiT PPI system (Promega, Madison, WI) as a cell-based orthogonal assay to test the hits identified in the AC1/CaM FP pilot screen. The NanoBiT assay provides a cellular context to examine compound activity and utilizes full-length adenylyl cyclase. We have applied this system in a prior study to assess the AC8/CaM PPI (Hayes et al., 2018). In the NanoBiT system, proteins are tagged on their N- or C-terminus with large or small BiT (LgBiT or SmBiT) fragments. As the proteins associate in cells, the BiT fragments complement to form a functional luciferase enzyme (NanoLuc) capable of producing luminescence when the substrate (NanoGlo reagent) is present. As the two interacting proteins can be tagged with the LgBiT or SmBiT on their N- or C-termini, there are eight total NanoBiT vector combinations for a PPI pair. Further, the relative sizes of the BiT fragments, with LgBiT at 17 kD and SmBiT at only 11 amino acids, necessitate testing of each NanoBiT vector combination to assess any impact the BiT fragment presence has on the PPI due to steric hindrance (Supplementary Figure S3). These vectors were generated as described above, and transient transfection methods were used to express the vector pairs in HEK293T cells. In addition to the vectors listed above, a Halo-SmBiT control was used as a non-specific protein to establish baseline luminescence from LgBiT activity, which is typically negligible in the absence of the SmBiT (168). The Ca^2+^-dependent nature of the AC1/CaM PPI allowed use of controls for the NanoBiT assay which modulate the level of intracellular Ca^2+^. The AC1/CaM PPI response to calcium mobilizers was assessed for all vector combinations. Several assay conditions were used to determine the most promising vector combinations under varying levels of intracellular Ca^2+^. Briefly, BAPTA-AM was added 30 min before the addition of the NanoBiT substrate (NanoGlo Reagent) to enter cells and chelate free Ca^2+^. After this initial treatment, NanoBiT substrate was added, and baseline luminescence was measured continuously for 30 min. Finally, cells were treated with buffer, thapsigargin, or calcium ionophore A23187, and luminescence was monitored continuously for an additional 60 min. The response to vehicle or Ca^2+^ regulating mobilizing agents (Thapsigargin or A23187) in the absence or presence BAPTA-AM was assessed using the area under the curve (AUC) for the 25 min following the baseline luminescence read. Initial optimization efforts revealed poor results for the combinations with LgBiT attached to AC1 and SmBiT attached to CaM. This result is likely either the result of the LgBiT occluding CaM binding to the AC1 protein or the BiT fragments being too distant to associate and form functional NanoLuc enzyme. These Ca^2+^ sensitivity experiment results prompted us to use SmBiT-AC1 with LgBiT-CaM (both N-terminal attachments) to assess our initial hits using NanoBiT. This vector combination showed the most robust increase in luminescence with thapsigargin compared to A23187. In addition, the SmBiT-AC1 and LgBiT-CaM fusion protein combination was responsive to thapsigargin in terms of reversal of the decrease in luminescence from pre-incubation with BAPTA-AM **(** Supplementary Figure S3B).

### NanoBiT assay: AC1/CaM FP hits in cells

**FIGURE 5:**
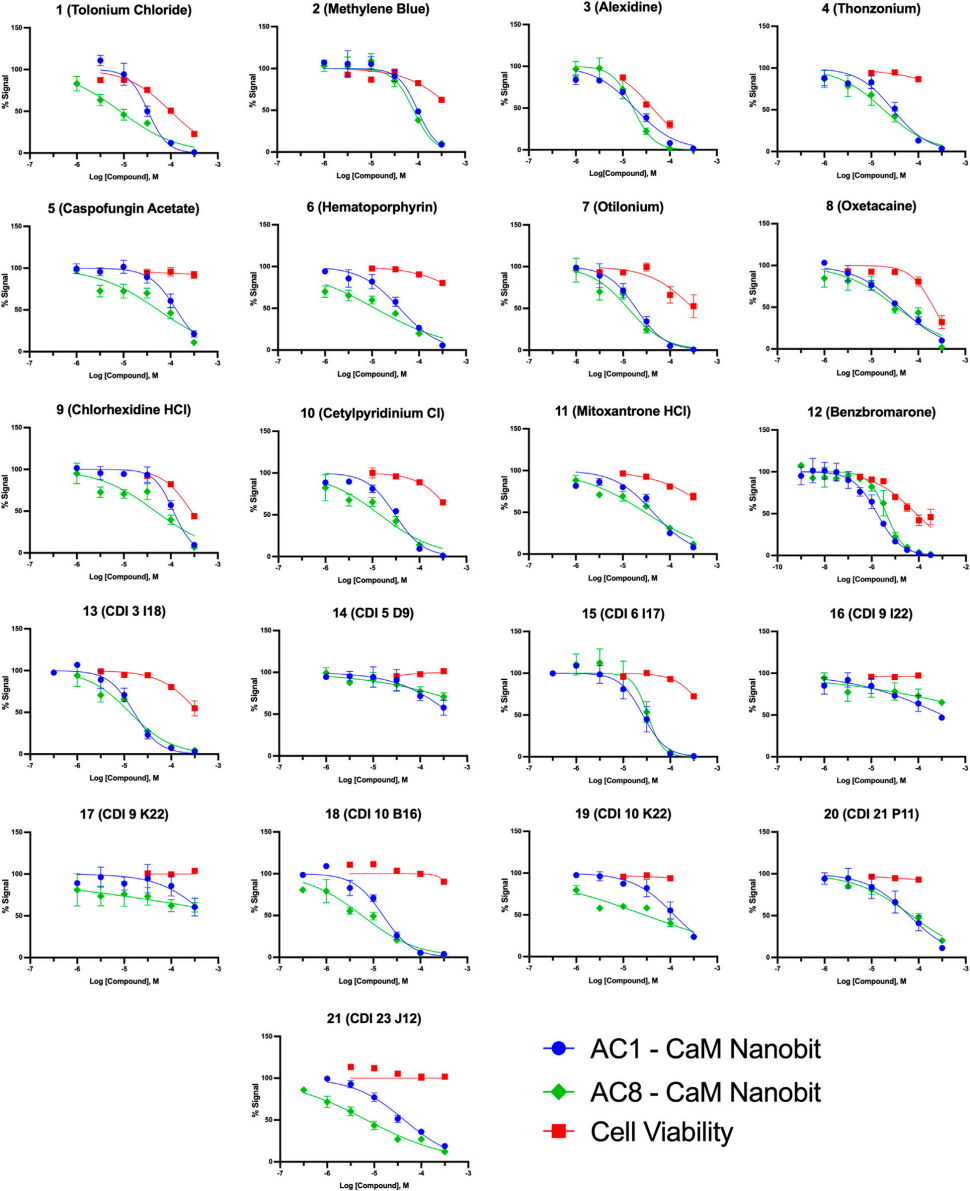
Concentration response curves for AC1/CaM pilot screen hits in NanoBiT assay and CellTiter Glo-2.0 cell viability assay. NanoBiT results are shown as blue circles for AC1/CaM, green squares for AC8/CaM, and red squares for cell viability (CellTiter Glo-2.0) assay results. The baseline corrected AUC values are normalized to vehicle in the presence of thapsigargin (100%) or vehicle pre-treated with BAPTA-AM (0%) for the NanoBiT assay. The cell viability data is normalized to the cells treated with vehicle (100%) or cell free wells with vehicle added (0%). Data represents n = 3 experiments ±SEM.

Pairing the NanoBiT assay with the Cell-Titer Glo 2.0 assay allowed us to monitor a decrease in the NanoBiT signal and ensure this signal loss was not a result of cell death or loss of membrane integrity. The CRCs for hit compounds in the NanoBiT and cell viability assays are shown in Figure 5 and summarized in Table 4. One of the most promising hit compounds was **12** (Benzbromarone). In the NanoBiT assay, **12** was the most potent hit with an IC_50_ of 1.70 ± 1.17 μM and 4.53 ± 1.15 μM for the AC1/CaM and AC8/CaM PPI, respectively. Cell viability at the AC1/CaM IC_50_ was at 90% for **12**. Despite performing well in the NanoBiT assay, **12** was not the most potent hit in the AC1/CaM FP assay with an IC_50_ of 33 μM and a K_i_ of 7.8 μM. In the FP assay, **12** was approximately 7- and 3-fold selective for AC1-C1b/CaM over AC8-Nt/CaM and AC8-C2b/CaM, respectively. This selectivity is not entirely lost in the NanoBiT assay but is a theme observed for several hit compounds tested.

**TABLE 4:** Results for AC1/CaM pilot screen hits in the NanoBiT and cell viability assay. Data are reported as IC_50_ values with standard error of the mean for the AC1/CaM and AC8/CaM NanoBiT assays. All data represent the average ±SEM of three individual experiments The final column shows the % cell viability in the presence of each compound at it is AC1/CaM NanoBiT IC_50_ concentration, using the CellTiter Glo-2.0 cell viability assay as previously described. All data represent the average ±SEM of three individual experiments.

#	Compound	Nanobit: AC1-CaM IC_50_ (μM ± SEM)	Nanobit: AC8-CaM IC_50_ (μM ± SEM)	% Viable Cells at AC1 IC_50_
**1**	Tolonium Chloride	32.9 ± 1.01	8.4 ± 1.19	80
**2**	Methylene Blue	98 ± 1.14	80.3 ± 1.14	86
**3**	Alexidine	17.9 ± 1.15	16.5 ± 1.15	75
**4**	Thonzonium	29.1 ± 1.2	18.5 ± 1.26	100
**6**	Hematoporphyrin	38.4 ± 1.17	11.6 ± 1.24	95
**7**	Otilonium	18.8 ± 1.16	12.8 ± 1.22	96
**8**	Oxetacaine	40 ± 1.15	34.6 ± 1.32	93.3
**9**	Chlorhexidine	111 ± 1.23	47.1 ± 1.35	80
**10**	Cetylpyridinium Cl	30.4 ± 1.1	14.4 ± 1.29	96
**11**	Mitoxantrone	44 ± 1.15	29.8 ± 1.18	87
**12**	Benzbromarone	1.7 ± 1.17	4.16 ± 1.15	90
**13**	CDI 3 I18	16.1 ± 1.13	13.1 ± 1.19	96
**14**	CDI 5 D9	84 ± 1.7	N/A	100
**15**	CDI 6 I17	26.1 ± 1.17	33.3 ± 1.18	100
**18**	CDI 10 B16	16.1 ± 1.12	5.71 ± 1.22	100
**20**	CDI 21 P11	63 ± 1.34	70.3 ± 1.13	100
**21**	CDI 23 J12	25.6 ± 1.42	6.8 ± 1.19	100

The next most potent hits in cells were **3**, **7**, **13**, and **18,** with IC_50_ values below 20 μM for the AC1/CaM and AC8/CaM NanoBiT assays. These compounds were moderately selective for AC1 in the FP assays, but selectivity was lost in the NanoBiT assay. Interestingly, the AC selectivity of **18** flipped from what was observed in the FP assay, where **18** was selective for AC1-C1b/CaM over both the AC8/CaM FP constructs; in the NanoBiT assay, **18** was ∼3-fold selective for AC8 over AC1. This phenomenon was not specific to **18**. For example, the hit compounds **6** and **21** were 2- to 4-fold selective for AC8 over AC1 in the NanoBiT assay but exhibited near total selectivity for AC1-C1b/CaM over the AC8/CaM constructs in the FP assays. We will discuss possible explanations for this reversal of selectivity below.

With respect to cell viability, amongst all the pilot screen hits, **3** had the greatest decrease in cell viability of 25% at its AC1/CaM IC_50_ (17.9 ± 1.15 μM) and 70% at 100 μM. For hits **7**, **13,** and **18,** cell viability was between 96–100% at their respective AC1/CaM IC_50_ values. For **7** and **13**, cell viability was reduced by ∼50% at the highest concentration tested (316 μM), but 90% cell viability was observed for **18** at this concentration. From the remaining pilot screen, only hits **1**, **2**, **9**, and **11** exhibited a reduction in cell viability greater than 10% at their respective AC1/CaM IC_50_ values. Although **1** was the most potent in the FP assays, this was not the case in the NanoBiT assay. **1** was more potent in the AC8/CaM NanoBiT assay, with IC_50_ values of 8.4 and 32.9 μM in the AC8 and AC1/CaM NanoBiT assay, respectively. The remainder of the pilot screen hits tested *in vitro* had AC1/CaM IC_50_ values between 20 and ∼100 μM or did not inhibit the AC/CaM PPI in the NanoBiT assay. For simplicity, the remaining sets were separated by a range of IC_50_ values from the AC1/CaM NanoBiT assay: Set A—IC_50_ values from 1 to 20 μM: **3**, **7**, **12**, **13**, and **18**; Set B—IC_50_ values from 21 to 30 μM: **4**, **15**, and **21**; Set C—IC_50_ values from 30 to 40 μM: **1**, **6**, and **10**; Set D—IC_50_ values from 41 to 150 μM: **2**, **5**, **8**, **9**, **11**, **14**, and **20**; Set E—No Inhibition in NanoBiT assay: **16**, **17**, and **19.**

### Nuclear magnetic resonance: CaM binding

**FIGURE 6:**
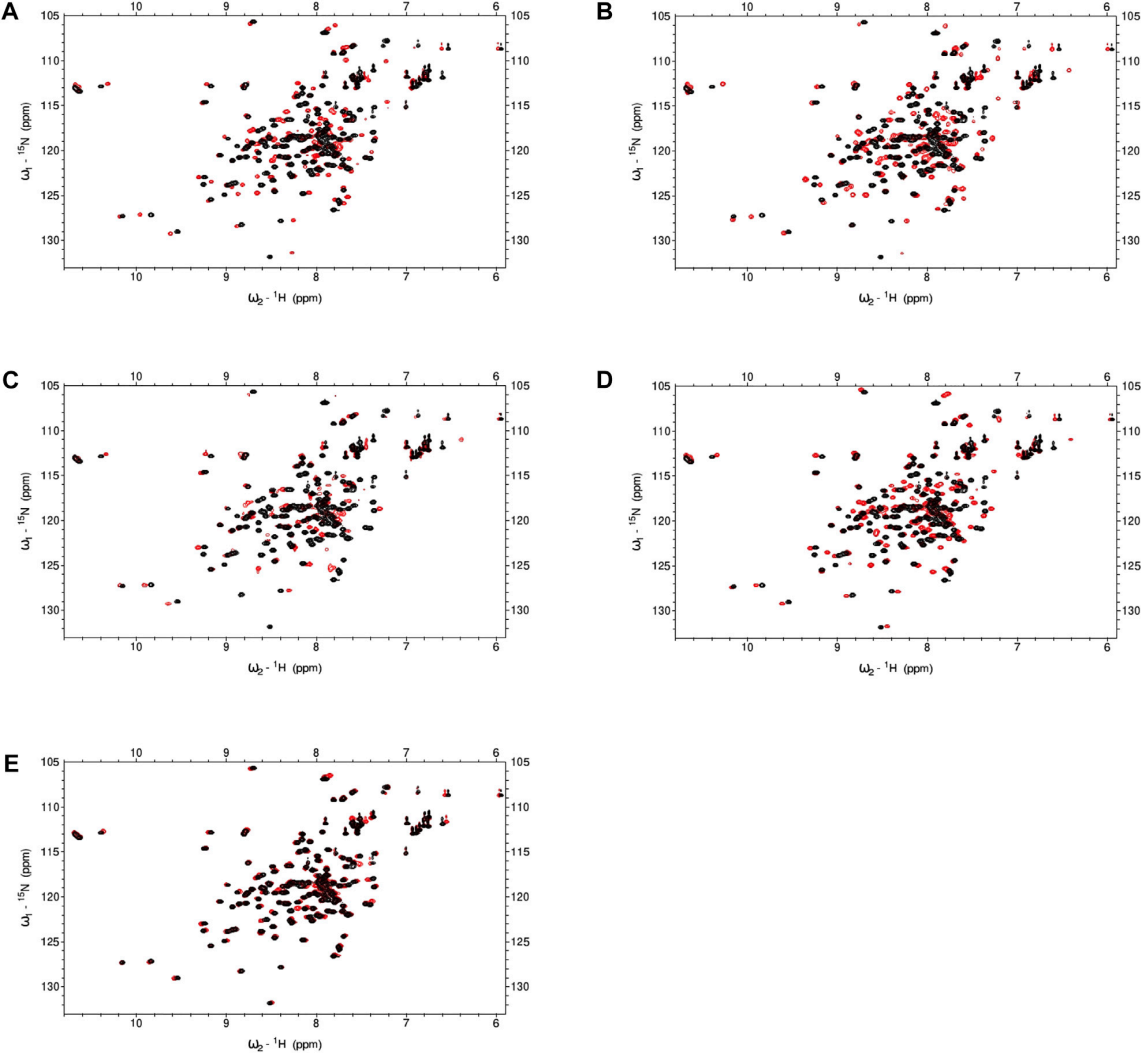
NMR spectra for CaM in the presence or absence of hit compounds. **(A–E)** ^1^H,^15^N-HSQC for 100 μM ^15^N-CaM with 10 mM Ca^2+^ in the absence or presence (red) of hit compounds at 5EQ molar excess over CaM. **(A)** Hit **12 (B)** Hit **13 (C)** Hit **15 (D)** Hit **18 (E)** Hit **21**. Buffer: 100 mM KCl, 20 mM HEPES pH7.4, 10 mM CaCl_2_, 10% D_2_O, 5% DMSO, Temperature: 298 K.

To ascertain if certain compounds were imparting inhibition through direct binding to calmodulin, we collected ^1^H-^15^N HSQC spectra of ^15^N-CaM in the presence or absence of the compound. We tested hit compounds **12**, **13**, **15**, **18**, and **21** at 5 equivalent molar excess of CaM concentration and observed chemical shift perturbations (CSP) for multiple CaM residues (Figure 6). Ligand-induced CSPs indicate binding as observed by backbone amides on ^15^N-CaM. Hits **12** and **13** exhibited the largest CSPs in the AC1 set with CaM likely to be fully saturated or in fast exchange at 5EQ molar excess over CaM. Hits **18** and **21** exhibit modest chemical shift changes at 5EQ. Only compound **15** exhibited chemical shift changes where several peaks were broadened by intermediate exchange and disappeared. The disappearance of peaks in intermediate exchange occurs near the equilibrium between free and bound states that are on a time scale typical for conformational dynamics. At 5EQ molar excess of **15**, CaM is not fully saturated. The somewhat unique binding properties of **15** to CaM may provide opportunities for AC1 vs. and AC8 selectivity.

### Characterization of FP and NanoBiT hits in AC1 cAMP accumulation assays

From the hits identified among FP screens and NanoBiT assays, six were selected for characterization of activity against AC1, based on their potency and selectivity profiles as well as favorable drug-like properties. Hits from the CDI library (**12**, **13**, **15**, **18**, **20**, and **21**; Table 5) were AC1-selective in the FP assays with moderate efficacy and displayed modest potency against AC1 and/or AC8 in cellular NanoBiT assays. Hit **12** was identified in the AC1 FP screens but was not identified as a hit against AC8, indicating possible selectivity for AC1. Furthermore, **12** displayed the highest potency at inhibiting the PPI between AC1-CaM and AC8-CaM in NanoBiT assays. For initial characterization of these six compounds, we utilized HEK-293 cell lines where endogenous AC3 and AC6 were knocked out to reduce the background cAMP signal and subsequently transfected with AC1 (AC1-HEK Δ3/6 KO cells; (Soto-Velasquez et al., 2018). cAMP accumulation is measured as a direct output of AC inhibition via a Cisbio homogenous time-resolved fluorescence (HTRF) assay. The data shown in Table 5 and represent the mean ± SEM of the IC_50_ values (µM) for each compound, in AC1-HEK Δ3/6 KO cells. Hit **12** displayed the highest potency against AC1 at 4.09 μM, with **15** following closely at 4.89 μM; both of these compounds were equally efficacious, displaying ∼75% inhibition of AC1 activity (data not shown). Hits **18** and **21** were slightly less potent at 6.70 and 8.77 μM, respectively. **18** and **21** also displayed lower efficacies, at ∼70 and ∼50%, respectively. Finally, hits **13** and **20** were significantly less potent at AC1 than other hits evaluated, at 31.0 and 66.3 μM, respectively.

**TABLE 5:** Initial examination of selected hits against Ca^2+^-stimulated AC1 activity in cAMP accumulation assays. AC1-HEK Δ3/6 KO cells plated at 10,000 cells per well. Compounds were added to wells and cAMP accumulation was stimulated with 3 μM A23187 for 1 h at RT. IC_50_ values ±SEM generated from 3-parameter nonlinear regression from three independent experiments (N = 3).

Compound #	AC1 IC_50_ ± SEM
12	4.09 ± 2.41
13	31.0 ± 6.63
15	4.88 ± 2.14
18	6.70 ± 0.70
20	66.3 ± 33.3
21	8.77 ± 4.47

### Structure-activity relationship (SAR) studies of dithiophene CDI compounds in AC1/AC8 cAMP accumulation assays

**FIGURE 7:**
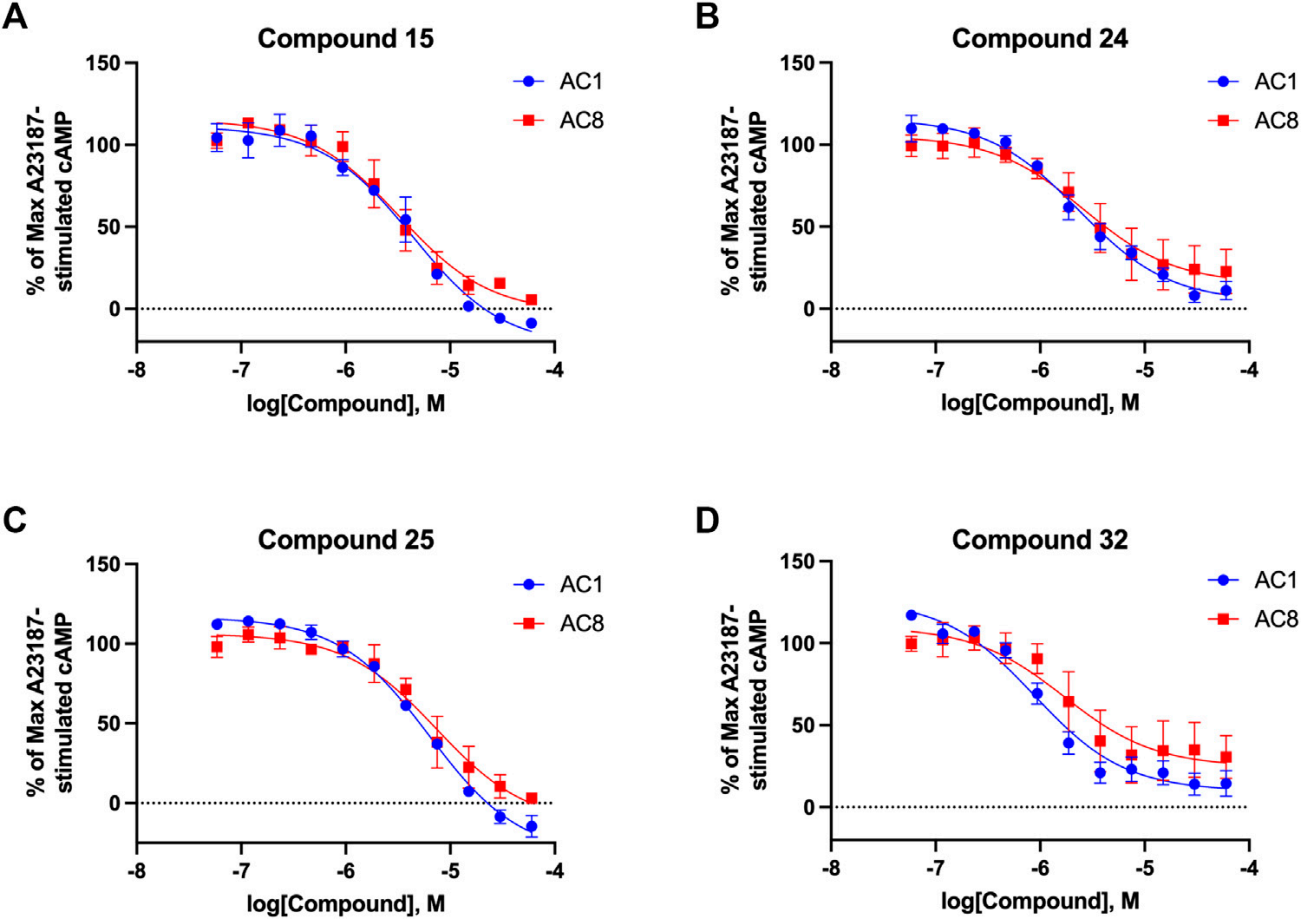
cAMP accumulation CRCs of CDI compounds at AC1 and AC8. cAMP accumulation data of select CDI compounds 15 **(A)**, 24 **(B)**, 25 **(C)**, 32 **(D)**, in AC1- or AC8-HEK Δ3/6 KO cells. Points in representative summary curve displays the mean ± SEM of the percent of 3 µM A23187 inhibition from the averages of three independent experiments (N = 3).

Among the hits that were identified and characterized for AC1 activity inhibition, the dithiophene scaffold of compound **15** from the CDI library was prioritized for initial SAR evaluation for several reasons: 1) the combined potency and selectivity in the FP assays, 2) efficacy for PPI inhibition in the NanoBiT assay, and 3) drug-like physicochemical properties. To accomplish this, we utilized HEK Δ3/6 KO cells expressing AC1 or AC8. The data shown in Table 6 represent the mean ± SEM of the IC_50_ values (µM) for each compound, in AC1- and AC8-HEK Δ3/6 KO cells, respectively. Testing of the dithiophene compounds, referred to hereafter as CDI analogs (Table 6), with A23187-stimulated AC1 and AC8 activity yielded a unique SAR profile, focused on the cyclic amine ring of the dithiophenes as well as substituents from this ring and to the thiophene heterocycles. For this assessment, 12 additional CDI analogs (**22–33**) were purchased from ChemDiv, along with the original dithiophene hit **15**. For the first piperidine series (**22–24**), SAR was relatively flat with modest changes to either the piperidine or alkyl group on the thiophene leading to equipotent analogs. **24** was the most potent of this cohort versus AC1 with an IC_50_ value of 2.75 μM. This molecule also was equipotent against AC8 with an IC_50_ values of 2.70 μM. Moving from a piperidine to the matched molecular pair *N*-alkylated piperazine analogs (**15, 25, 26**) provides a slight reduction in AC1 activity (2—3-fold) compared to the piperidine containing counterpart. The piperazine modification also had varying effects on AC8, particularly with the ethyl-thiophene derivatives (**15, 26**) slightly favoring AC8 over AC1. The next two analogs (**27, 28**) contained a fused cyclic alkane to the central thiophene of the scaffold, as opposed to methyl or ethyl substitution. Each exhibited marked reduction of activity against both AC1 and AC8 compared to nearest neighbor analogs non-cyclic analog. The reduction was greater against AC1, thus making these analogs more selective for AC8, with **28** being 2-fold more potent at AC8. In both AC1- and AC8-overexpressing HEK cells, several of these compounds displayed approximately 100% inhibition of cAMP accumulation, as well as low-micromolar IC_50_ values at both AC1 and AC8. **29** maintained the *N*-methylpiperazine and ethyl substituent off the central thiophene, but swapped the second thiophene heterocycle for a phenyl ring. When compared to its matched molecular pair in **15, 29** exhibited about a 2- and 3-fold reduction in AC1 and AC8 potency, respectively. **30–32** swapped the piperazine for a morpholine moiety. Notably, this modification decreased both AC1 and AC8 potency for the matched molecular pairs that incorporated either the piperazine or piperidine with one exception. **32** actually displayed an improved potency against A23187-stimulated cAMP accumulation in AC1-HEK cells with an IC_50_ at AC1 of 0.89 µM. This molecule was also modestly selective over AC8 with an IC_50_ of 1.86 µM. Interestingly, although **32** is slightly more potent against AC1, the AC8 potency makes this compound the most potent AC8 inhibitor known to date. The final analog in this SAR set was **33**, which moved from the *N-*cyclic alkane substituent and incorporated a 2-aminopyridine. This compound displayed similar potency to the piperidine series (**22–24**), with modest selectivity for AC8. Concentration-response curves for selected analogs (**15**, **24**, **25**, and **32**) are presented in Figure 7. Among these four compounds are some of the most potent inhibitors at AC1 and AC8, also displaying 100% inhibition of AC1 activity and 70–100% inhibition of AC8 activity (Table 6 and Figure 7).

**TABLE 6:** CDI analog structures, IC_50_ values from cAMP accumulation assays in AC1- and AC8-HEK Δ3/6KO cells, and cell viability in AC1-HEK Δ3/6KO cells. IC_50_ values were generated from a 3-parameter nonlinear regression of the averages of 3 independent experiments (N = 3).

#	Structure	IC_50_ (μM) ± SEM		% Viable Cells at AC1	#	Structure	IC_50_ (μM) ± SEM		% Viable Cells at AC1
15	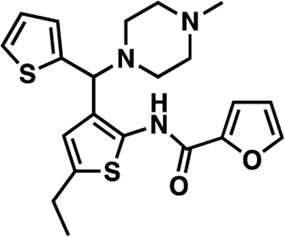	**AC1**	**AC8**	**30 μM**	**28**	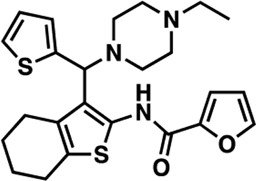	**AC1**	**AC8**	**30 μM**
		4.36 ± 0.32	3.90 ± 1.83	43			20.0 ± 5.23	11.6 ± 1.82	88
22	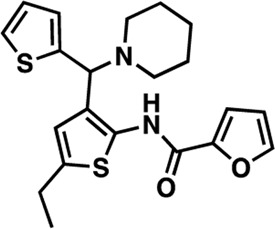	2.48 ± 0.39	3.23 ± 0.57	97	**29**	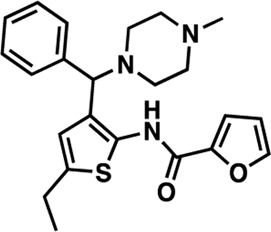	10.3 ± 1.45	14.4 ± 4.10	65
23	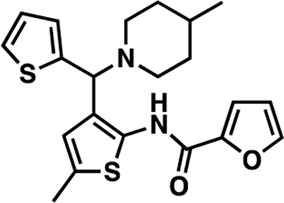	3.08 ± 0.25	4.21 ± 1.01	100	**30**	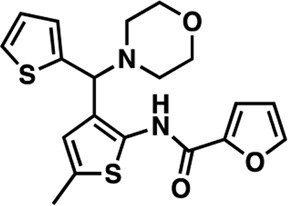	10.6 ± 2.72	4.99 ± 1.10	100
24	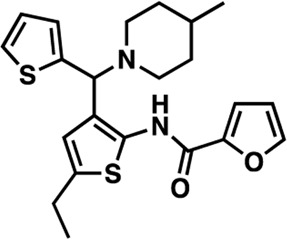	2.75 ± 0.82	2.70 ± 0.24	77	**31**	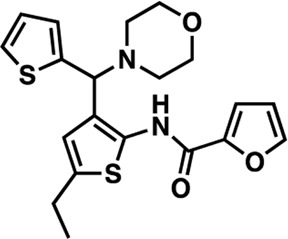	5.25 ± 1.07	1.49 ± 0.38	37
25	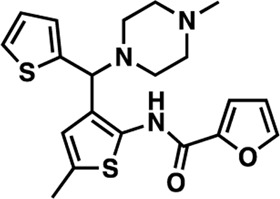	6.39 ± 0.89	8.85 ± 2.70	94	**32**	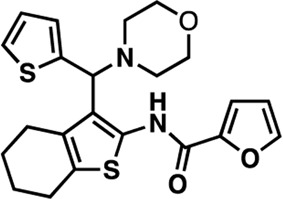	0.89 ± 0.04	1.86 ± 0.32	100
26	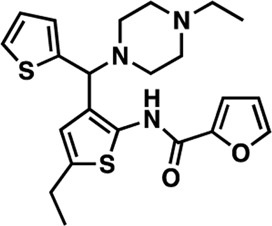	4.39 ± 0.44	2.92 ± 1.36	61	**33**	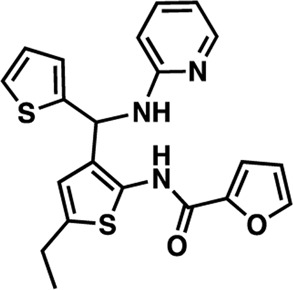	3.58 ± 2.26	2.70 ± 1.92	88
27	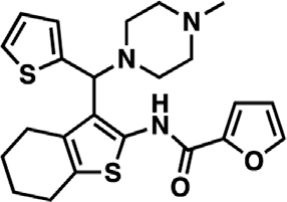	34.8 ± 13.0	25.6 ± 6.77	48					

### Isoform selectivity of CDI compounds at AC2 and AC5

To determine whether these compounds were selective for AC1 and AC8 over other isoforms, we tested **15**, **24**, **25**, and **32** in cAMP assays against AC2 and AC5 as representative isoforms from Groups II and III, respectively, of AC isoforms. We used AC2-HEK Δ3/6 KO and AC5-HEK Δ3/6 KO cells and tested each of the four compounds at 30 μM, assessing percent inhibition, shown in Table 7. Surprisingly, hit compound **15** displayed modest inhibition at AC2 and AC5 (59 and 47%, respectively). Compound **25** also partially inhibited AC2 and AC5 activity (52 and 46%, respectively). **15** and **25** are structurally almost identical, differing only in the substituent on the central thiophene (ethyl on **15** versus methyl on **25**), which may explain why these had similar activity against AC2 and AC5. On the other hand, compounds **24** and **32**, having a piperidine and morpholine heterocycle, respectively, showed very little inhibition against AC2 and AC5 (<16%). Interestingly, in our previous studies we have observed AC1 inhibitors to modestly potentiate AC2 at 10 and 30 μM, thus, the inhibition at 30 µM was a bit unexpected but may be a result of the different mode of action for this scaffold. This will be investigated in future studies.

**TABLE 7:** Percent inhibition of CDI compounds (30 μM) in AC2-HEK Δ3/6KO and AC5-HEK Δ3/6KO cells. Data reported as averages of % inhibition values at 30 μM from three independent cAMP accumulation assays (N = 3) ± SEM.

Compound #	% Inhibited at AC2 ± SEM	% Inhibited at AC5 ± SEM
15	59 ± 14	47 ± 10
24	15 ± 11	16 ± 11
25	52 ± 11	46 ± 5
32	10 ± 8	3 ± 3

## Discussion

The FP assay developed for this work allowed us to measure the interaction between CaM and an AC1-C1b peptide. In our FP assay, we utilized peptides labeled with the fluorescent dye cyanine-5 (Cy5) that incorporated the residues on AC1 or AC8 where Ca^2+^ dependent CaM binding has been previously established (Masada et al., 2012; Herbst et al., 2013). In the AC-CaM FP assays, the Cy5-AC peptides are approximately 4 kD (AC1-C1b: 3,868 g/mol, AC8-Nt: 3,746 g/mol, AC8-C2b: 3,549 g/mol) and GST-CaM is 39 kD (His6-GST: 25 kD, CaM: 14 kD). The addition of the GST-tag to CaM improved the degree of polarization for the AC peptide when it was bound to GST-CaM and increased the FP signal window necessary for HTS. After our optimization efforts, the AC1/CaM FP assay proved to be robust, evidenced by Z’ values ranging from 0.68–0.77 throughout the compound screen, consistently above the lower threshold of 0.5 for assays to be considered robust and suitable for HTS hit identification (Zhang et al., 1999). Further, the assay tolerated up to 2.5% DMSO and was stable from 1 to 6 h (Supplementary Figure S1). Therefore, we prioritized optimizing our HTS approach to incorporate assays that were sensitive to Ca^2+^, a key mediator of the AC/CaM interaction. We were pleased to find that the interaction between CaM and the Cy5 labeled AC peptides was dependent on Ca^2+^ in the FP assays, as evidenced by the response observed when EGTA was present in the assay (Figure 1B). A drawback to this approach was the potential for metal chelators; we, therefore, assessed compounds that contained structures or exhibited inhibitory behavior that suggests the ability to chelate Ca^2+^. Our optimization process centered around our goal of identifying compounds that would perform well in a calcium-rich environment where the PPI occurs.

Approximately 21,000 compounds from 4 different chemical libraries were screened using the AC1/CaM FP assay. The pilot screen identified 54 compounds (FDA-Approved: 7 hits, MMSP: 13 hits, DIVERSet: 2 hits, and ChemDiv: 32 hits). However, due to library duplication, three compounds (Alexidine, Otilonium, and Thonzonium) were identified as hits in the FDA-Approved and MMSP libraries. An additional three compounds, Chicago Sky blue, Protoporphyrin IX, and Chlorophyllide Cu complex, were removed after their chemical structures were unfavorable for further development. The availability of ChemDiv CNS library stocks permitted initial concentration-response curve assessments to be made before ordering powder stocks. From the original 32 ChemDiv hits, 9 were advanced to the next phase of screening based on their approximate IC_50_ and percent inhibition relative to the positive control (100 μM CDZ = 100% inhibition) in the AC1/CaM FP assay. In addition to the reduction in hits from the CDI library (32–9), five compounds identified in the FDA-approved MMSP and ChemBridge library screens did not exhibit significant inhibition of AC1/CaM in the FP assay after fresh powder stocks were ordered. Two of those compounds were from the ChemBridge DIVERSet library, two were from the Microsource spectrum collection (ethyl quinine and rivastigmine tartrate), and one was from the FDA-approved library (nicotine ditartrate). Compounds that exhibited poor potency or inhibition in the AC1/CaM FP assay were tested in the AC8/CaM FP assays to gather information about what chemical scaffolds/structures tended to be more selective for the AC8/CaM interaction(s) over the AC1/CaM interaction. As a result, 25 of the original 54 hit compounds were advanced for further characterization.

Characterization of hits centered around CRC analysis of the primary AC1/CaM FP assay, with counter screening efforts to identify hit compounds that selectively inhibited the AC1/CaM interaction over the AC8/CaM interaction(s). The two most potent hits identified in the FP pilot screen, **1** and **2**, contain a phenothiazine structure. These hits have the same phenothiazine scaffold as the known CaM antagonist trifluoperazine (TFP). This common substructure could indicate that the AC/CaM inhibition observed for **1** and **2** results from CaM binding rather than AC binding. However, TFP poorly inhibited AC1/CaM in the FP assay with only 30% inhibition at 100 μM for AC1/CaM. Further, our prior testing with TFP in the AC8/CaM FP assays found that it could inhibit AC8-Nt/CaM (∼85% at 100 μM) but did not inhibit AC8-C2b/CaM (3). Despite their structural similarities, **2** has been found to reduce pain caused by cancer treatment as an oral rinse, alleviate the pain that accompanies a propofol injection, and reduce lower back pain as an intradiscal injection (Salman et al., 2011; Kallewaard et al., 2016; Roldan et al., 2017). Although the mechanism of **2** is not fully understood, the predominant proposed mechanisms involve the inhibition of monoamine oxidase A, nitric oxide synthase, guanylate cyclase (GC), and blockade of the GABA receptor (Alda, 2019; Bistas & Sanghavi, 2020). Acting in the CNS on a GC would suggest **2** could reach AC1 at the very least. However, this finding prompts further structural optimization of compounds with a phenothiazine scaffold to avoid off-target effects. Another structural feature shared by several hits (**3–5, 7**, **9**, and **10**) is a long carbon chain or linker (6 carbons or more). As these linkers will afford flexibility to these compounds, it may be possible that these aliphatic “tails” allow for more significant inhibition by binding to a hydrophobic pocket. However, further studies examining the binding site would need to confirm this notion. Apart from **20**, all the validated hits from the CDI library inhibited the AC1/CaM FP assay but could not inhibit both AC8/CaM FP assays. However, the hits from the CDI library exhibited a lower degree of inhibition in the AC1/CaM FP assay as compared to the hits from the FDA-approved or the MMSP libraries.

Of the 25 compounds, 21 were advanced for testing in NanoBiT assays. The goal of this AC1/CaM FP HTS was to identify novel structures capable of disrupting the AC1-C1b/CaM and AC1/CaM interactions. In pursuit of this goal, two assays were developed to detect this interaction in both a biochemical setting and in a living cellular environment. Screening ∼22,000 compounds from four unique chemical libraries has yielded small molecule inhibitors with interesting and novel chemical scaffolds that can disrupt the AC1/CaM interaction.

The NanoBiT assay was used an approach for assessing the ability of the hit compounds to inhibit the full-length AC/CaM proteins in a live cell format. As mentioned above, **12** was the most potent compound in the AC1/CaM NanoBiT assay. **12** (Benzbromarone) contains a 1-Benzfuran scaffold with an ethyl group substituted at the C-2 position and a 3,5-dibromo-4-hydroxybenzyl group at the C-3 position. Although **12** is labeled as a uricosuric agent and a known xanthine oxidase inhibitor, **12** was never approved by the FDA due to reports of acute liver toxicity (Chen et al., 2011; Chen et al., 2016). Compounds **1** and **2**, the most potent hits in the AC1/CaM FP assay, exhibited a significant loss of activity in the NanoBiT assay. Compounds **3** and **4**, which preferentially inhibited AC1 in the FP assays, displayed a reduced AC1 selectivity in the NanoBiT assays. Previous work has found that **3** and **4** exhibit CaM binding (Hayes et al., 2018). This observation could indicate that these compounds do not exert their AC1/CaM inhibition by binding to the C1b domain of AC1 but rather bind to CaM. While this mechanism may challenge AC selectivity, the interactions of CaM with AC1 and AC8 are not identical, and thus binding to CaM does not entirely undermine the ability of compounds to be selective.

In the transition from biochemical to cell-based assays, a loss in potency is not uncommon. Further, as the AC/CaM PPI is occurring not at the cell surface but inside the cell, a loss in potency could be attributed to a compounds inability to cross the cell membrane or a high degree of non-specific protein binding (Strelow et al., 2004). Finally, mechanistic insights into the AC/CaM interaction provide an additional rationale for compounds exhibiting a loss in potency when transitioning from the peptide-based FP assay to the NanoBiT assay. The NanoBiT assays assessed the ability of the compounds to inhibit the full-length PPI, which for AC8 incorporates two CaM binding domains that are believed to work in unison. Although the activity of both AC1 and AC8 is stimulated by Ca^2+^/CaM binding, their sensitivity to Ca^2+^ and binding mechanisms with CaM are distinct. For example, the activity of AC8 responds rapidly to transient fluxes of Ca^2+^ levels, while AC1 exhibits an increase in activity in response to Ca^2+^ flux that is sustained for a prolonged period (Masada et al., 2009; Masada et al., 2012). Although CaM has a higher affinity for AC1 relative to AC8, CaM must be fully saturated with Ca^2+^ before binding AC1 (Masada et al., 2009; Masada et al., 2012). On the other hand, the rapid response of AC8 to Ca^2+^ levels has been attributed in part to the presence of the two CaM binding domains, with a mechanism that is believed to involve tethering CaM to the N-Terminus of AC8 (Simpson et al., 2006; Masada et al., 2009; Masada et al., 2012). When Ca^2+^ levels increase, CaM is tethered to the N-terminus of AC8 and rapidly associates with the C2b domain of AC8, and an autoinhibitory mechanism is alleviated. Our FP approach does not fully capture this dynamic interplay between AC8 and CaM. As a result, several compounds exhibited a loss of selectivity for AC1 over AC8 in the NanoBiT assay relative to the FP assay. For example, the hits from the CDI library, apart from **20**, did not exhibit any degree of inhibition in either AC8/CaM FP assay, but were found to inhibit both the AC1 and AC8/CaM NanoBiT signal. Another aspect of the hits from the CDI library was their lack of toxicity, with only **13** and **15** exhibiting a decrease in cell viability at ∼300 μM.

Of the 21 compounds we advanced from the FP characterization, only 5 were advanced through further testing. The hits **12**, **13**, **15**, **18**, **20**, and **21** were selected based on their criteria as AC/CaM inhibitors in both the FP and NanoBiT assays. Although **3**, **4,** and **7** displayed similar potencies, these compounds have been assessed in a previous report (Hayes et al., 2018). Further, the selected hits contained chemical scaffolds amenable to further structural optimization.

As we characterized the hits from our FP screen, we began to focus on the unique chemotypes we identified in the process. Among the AC1-selective hits with regard to disruption of the AC1 peptide-CaM interaction in the FP assay was hit **15** (58% efficacy at AC1, IC_50_ = 22.1 µM). **15** was then confirmed as an AC1/AC8 non-selective hit against full-length AC1- and AC8-CaM in the NanoBiT assay (100% inhibition of both AC1- and AC8-CaM interactions; AC1-CaM IC_50_ = 26.1 µM, AC8-CaM IC_50_ = 33.4 µM). Compound **15** also had a number of favorable drug like properties (molecular weight = 415 g/mol and LogP = 0.53) and possessed multiple sites amenable to explore SAR. Lastly, we observed that compound **15** shared some structural overlap with a benzamide series of compounds that are known inhibitors of AC1 activity with selectivity vs. AC8 (Scott et al., 2022). We selected 13 dithiophene analogs including **15** for cellular testing against inhibition of Ca^2+^/CaM-stimulated AC1 and/or AC8 activity. This scaffold contains a central thiophene ring linked to an aromatic ring (furan) via an amide, which directly overlaps with the benzamide series (Scott et al., 2022). The central thiophene is also linked to a central carbon bound to the second aromatic ring (usually thiophene), with a variation of different heterocycles (piperazine, piperidine, morpholine, or pyridine). In our cAMP accumulation assays, we found that the piperazine analogs slightly reduced AC1 activity, 2- to 3-fold. We noticed a trend in which changing the methyl to an ethyl on the central thiophene caused the analog to be more selective for AC8. Cyclization of the alkyl substituent to the thiophene significantly reduced activity at both AC1 and AC8, and also shifted selectivity toward AC8. This suggests that more hydrophobic groups, or perhaps more rigidity, at the central thiophene may cause analogs to be more potent at AC8. Notably, combining the fused cyclohexane-thiophene ring system with a morpholine heterocycle significantly increased potency at both AC1 and AC8 (**32**). Importantly, **32** is the most potent inhibitor of AC8 activity known to-date, although this compound also potently inhibits AC1 activity. Overall, the limited SAR on this series suggests that AC1 and AC8 potency and selectivity can be tuned individually. AC1 activity was favored more by a methyl-substituent on the thiophene ring, whereas AC8 activity was favored more so by the ethyl in the same position. The cylcohexane-fused thiophene selectivity was highly dependent on the nature of the N-heterocycle with piperazine favoring AC8 activity and morpholine favoring AC1. These preliminary SAR data points will now inform design of future analogs to tune both AC1 and AC8 selectivity inhibitors from within the same series. As previously mentioned, some differences in potency and selectivity in cellular assays may be attributed to the differential interactions of AC1 and AC8 with CaM. Additionally, it is possible that other proteins within the cellular environment may affect the activity of these compounds.

Some degree of CaM binding was observed for all compounds tested in the NMR experiments. The ^1^H-^15^N HSQC spectra obtained for **12**, **18**, and **21** at 5EQ molar excess showed CaM was fully saturated or in fast exchange. To be in fast exchange, peaks exhibiting chemical shifts in the presence of a compound must move smoothly from free to bound (red) (Figure 6). In intermediate exchange, peaks will regain their original “free state” shape when saturation is reached but will exhibit broadened shape when in equilibrium between free and saturated, which was the case for **15**. Despite significant chemical shifts observed for **12** and **13**, we could not accurately predict a binding site on CaM. The abundance of residues exhibiting CSP made it difficult to accurately determine binding sites for these compounds without multidimensional NMR experiments, which was outside of the scope of this study.

Through development of a novel fluorescence polarization assay, screening of over 21,000 compounds for inhibition of AC1 and/or AC8, and validation of activity in novel NanoBiT assays, we have discovered a novel dithiophene scaffold. Interestingly, the dithiophene scaffold shares structural similarities with the AC1 inhibitor benzamide series (Scott et al., 2022). Unlike the benzamide series, however, many compounds among this small dithiophene set were not selective for AC1 over AC8. This may be attributed to differences in AC1- or AC8-CaM interactions, since CaM has one mode of interaction with the C1b domain of AC1, whereas it can interact with the N-terminal domain and the C2b domain of AC8. Although we screened and selected for AC1-selective inhibitors, it is possible that AC8 inhibition could be beneficial for anxiety associated with various chronic and inflammatory pain states in humans and rodents (see (Shiers et al., 2022) and references therein). Multiple studies have reported that AC8 knockout mice exhibited reduced anxiety-like behaviors in the elevated plus maze test (Schaefer et al., 2000; Bernabucci & Zhuo, 2016). Furthermore, Shiers et al. showed that *Adcy8* mRNA expression increased in the anterior cingulate cortex (ACC) in a mouse model of neuropathic pain, although AC8 knockout does not play a role in mechanical hypersensitivity after inflammation (Wei et al., 2002; Shiers et al., 2022). This suggests a potential role of AC8 in anxiety-like behaviors. Thus, a non-selective AC1/AC8 inhibitor may be desirable for treating both chronic pain and the associated anxiety. In future studies, it would be interesting to determine how these non-selective dithiophene compounds affect the interactions of AC1 and AC8 with CaM. Further characterization should also be done on these compounds, such as assessment of their activity against different AC activators such as Gα_s_ and forskolin. This dithiophene scaffold provides a novel AC1/AC8 inhibitor scaffold which is highly potent and may potentially be useful for treatment of chronic pain and anxiety.

## Data Availability

The raw data supporting the conclusions of this article will be made available by the authors, without undue reservation.
