# Supplementary figures and images for: Protein-protein interaction-based high throughput screening for adenylyl cyclase 1 inhibitors: Design, implementation, and discovery of a novel chemotype
Source: Front Pharmacol. 2022 Sep 6;13:977742. doi: 10.3389/fphar.2022.977742 (PMC9486168; doi:10.3389/fphar.2022.977742)

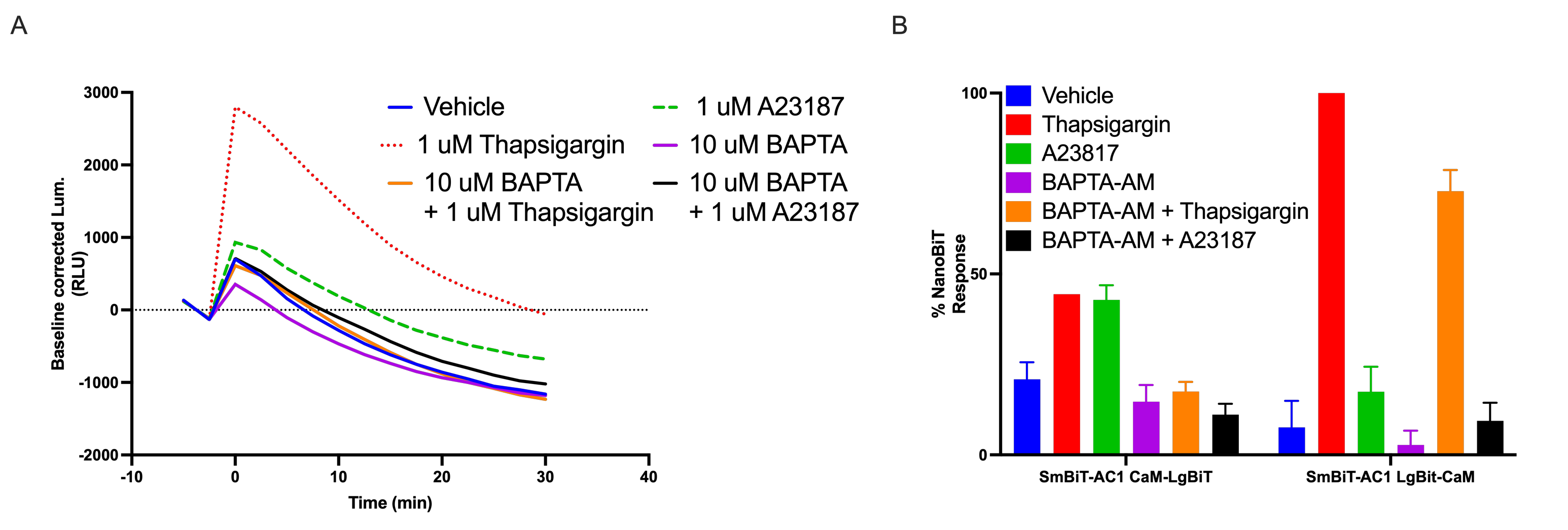

Supplement: Supplementary file 1 [file Image3.TIFF]

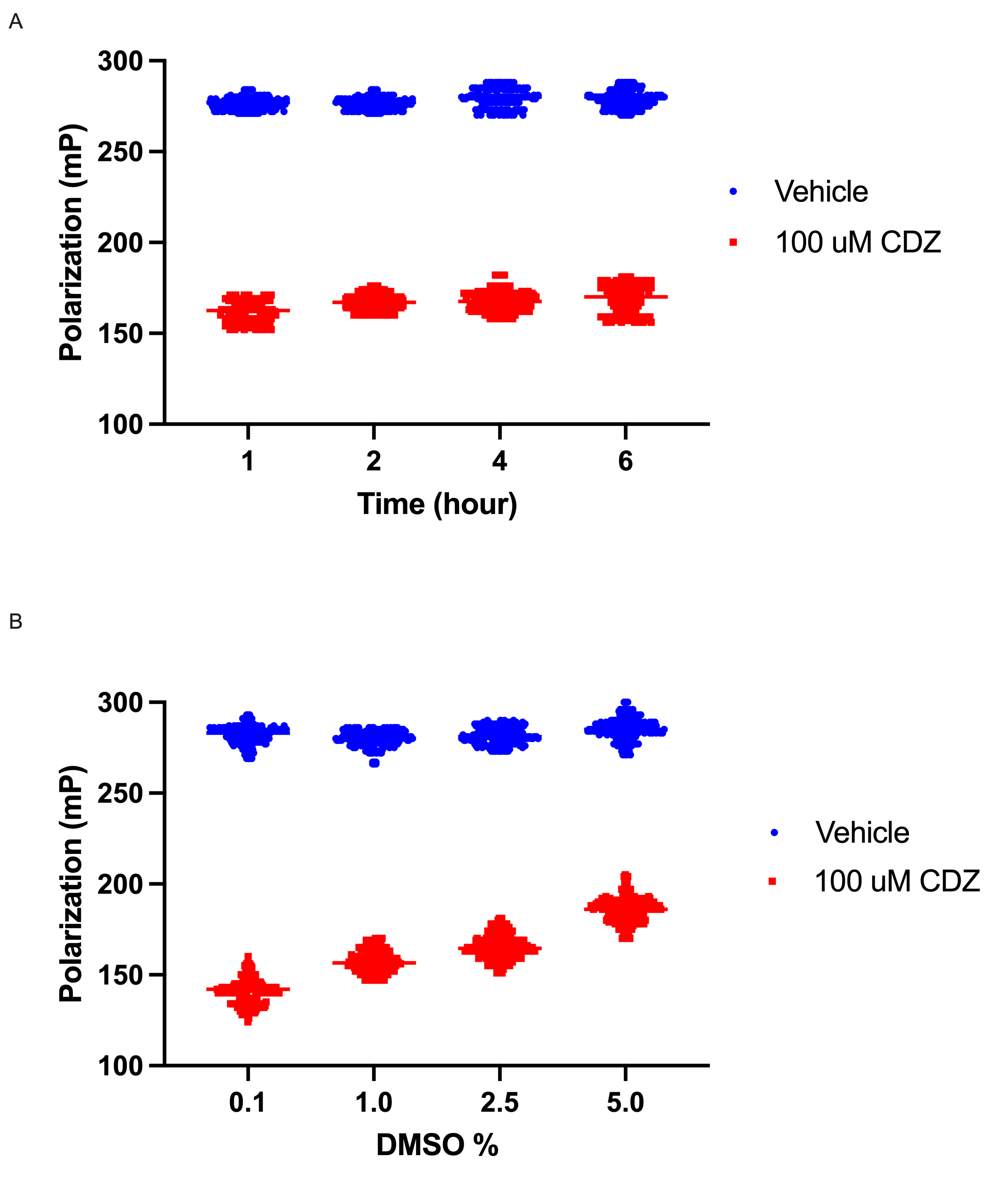

Supplement: Supplementary file 2 [file Image1.TIFF]

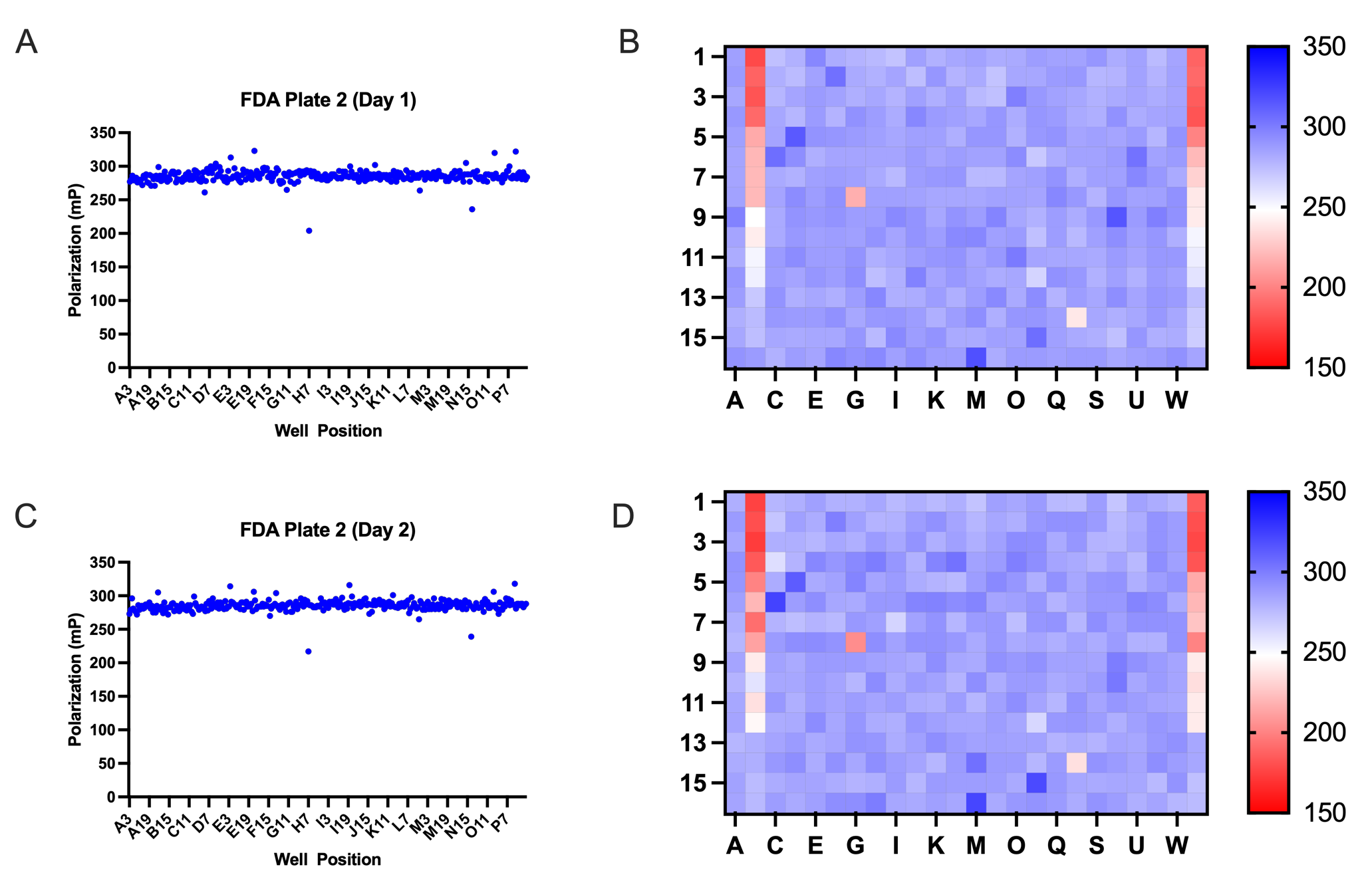

Supplement: Supplementary file 3 [file Image2.TIFF]
